# Activation of ChvG-ChvI regulon by cell wall stress confers resistance to β-lactam antibiotics and initiates surface spreading in *Agrobacterium tumefaciens*

**DOI:** 10.1371/journal.pgen.1010274

**Published:** 2022-12-08

**Authors:** Michelle A. Williams, Jacob M. Bouchier, Amara K. Mason, Pamela J. B. Brown

**Affiliations:** Division of Biological Sciences, University of Missouri-Columbia, Columbia, Missouri, United States of America; Max Planck Institute for Terrestrial Microbiology: Max-Planck-Institut fur terrestrische Mikrobiologie, GERMANY

## Abstract

A core component of nearly all bacteria, the cell wall is an ideal target for broad spectrum antibiotics. Many bacteria have evolved strategies to sense and respond to antibiotics targeting cell wall synthesis, especially in the soil where antibiotic-producing bacteria compete with one another. Here we show that cell wall stress caused by both chemical and genetic inhibition of the essential, bifunctional penicillin-binding protein PBP1a prevents microcolony formation and activates the canonical host-invasion two-component system ChvG-ChvI in *Agrobacterium tumefaciens*. Using RNA-seq, we show that depletion of PBP1a for 6 hours results in a downregulation in transcription of flagellum-dependent motility genes and an upregulation in transcription of type VI secretion and succinoglycan biosynthesis genes, a hallmark of the ChvG-ChvI regulon. Depletion of PBP1a for 16 hours, results in differential expression of many additional genes and may promote a stress response, resembling those of sigma factors in other bacteria. Remarkably, the overproduction of succinoglycan causes cell spreading and deletion of the succinoglycan biosynthesis gene *exoA* restores microcolony formation. Treatment with cefsulodin phenocopies depletion of PBP1a and we correspondingly find that *chvG* and *chvI* mutants are hypersensitive to cefsulodin. This hypersensitivity only occurs in response to treatment with β-lactam antibiotics, suggesting that the ChvG-ChvI pathway may play a key role in resistance to antibiotics targeting cell wall synthesis. Finally, we provide evidence that ChvG-ChvI likely has a conserved role in conferring resistance to cell wall stress within the Alphaproteobacteria that is independent of the ChvG-ChvI repressor ExoR.

## Author summary

Soil dwelling bacteria reside in changing environments requiring them to frequently adapt to stressful conditions to ensure survival. The bacterial envelope provides structural integrity and protection against osmotic stress and turgor pressure imposed by the environment. While the mechanisms of cell membrane and cell wall biogenesis have been extensively studied, our understanding of how diverse microbes respond to cell envelope and cell wall stress to increase their fitness remains limited. In this work, we identify ChvG-ChvI regulon as an envelope stress response system that confers protection under cell wall stress conditions in the bacterial plant pathogen *Agrobacterium tumefaciens*. This is a new function for the well-characterized ChvG-ChvI pathway which is also acid induced and promotes plant host invasion. Our results suggest that the ChvG-ChvI pathway has a broadly conserved role in protecting Alphaproteobacterial cells from extracellular stress and a more specific role in response to acid stress and promoting plant-microbe interactions.

## Introduction

The soil environment is constantly in flux and can undergo rapid changes in hydration, nutrient availability, temperature, acidity levels and many other abiotic and biotic factors [1]. To survive in these conditions, soil-dwelling bacteria must be able to monitor and respond to the changes around them. One of the main mechanisms bacteria employ to monitor changes in their environment is coupling environmental stimuli to transcriptional regulation using two-component systems (TCS) [2]. In turn transcriptional changes can modify bacterial behavior. In the plant-pathogen *Agrobacterium tumefaciens*, two TCS sense the presence of a potential host and initiate transcription programs that transition the bacterium into a virulent state [3].

The VirA-VirG histidine kinase/response-regulator pair is a characteristic of Agrobacteria and responds to plant phenolic compounds such as acetosyringone. Activation induces expression of the *vir* regulon, which encodes genes that are required for pathogenicity and plant transformation [4]. The ChvG-ChvI TCS is more broadly conserved across many Alphaproteobacteria but has been best characterized among the plant symbionts of Rhizobiales such as *Sinorhizobium meliloti* [5,6]. Activation of ChvG-ChvI is required for the transition from a free-living bacterium to a host-associated lifestyle [7]

In *S*. *meliloti* and *A*. *tumefaciens* ChvG-ChvI is regulated by the periplasmic protein ExoR. Under neutral conditions, ExoR binds to and represses ChvG; however, when cells are exposed to acidic conditions, ExoR is proteolyzed, which allows for activation of the ChvG-ChvI TCS [8,9]. ChvI induces transcriptional changes in many genes across several major pathways. For example, ChvI upregulates transcription of *mirA*, encoding a repressor of the motility response regulator Rem and ultimately resulting in suppression of genes for motility and chemotaxis [10]. ChvI also upregulates genes for exopolysaccharide production and, in *A*. *tumefaciens*, induction of the Type VI Secretion System (T6SS) [11]

Conservation of the ChvG-ChvI TCS is taxonomically constrained to several orders of Alphaproteobacteria, many of whom have free-living lifestyles that are never host-associated [6]. This begs the question: why is the ChvG-ChvI pathway conserved in so many non-host-associated bacteria? Recent interest in the ChvG-ChvI pathway of *Caulobacter crescentus* (ChvGI) provides a glimpse at the function of the pathway in the context of a bacterium with a drastically different ecological niche to that of *A*. *tumefaciens* or *S*. *meliloti*. ChvGI of *C*. *crescentus* senses and responds to osmotic stress and mutants of ChvGI are sensitive to several cell-wall targeting antibiotics [12,13]. It remains unclear if this function is solely a characteristic of *C*. *crescentus* ChvGI or if it is conserved across ChvG-ChvI orthologs.

Although the cell wall is an essential feature of bacteria that protects them from environmental stressors, relatively little is known about how bacteria sense and respond to changes in the composition of their cell wall. Peptidoglycan (PG) is a heteroglycan decorated with cross-linked peptide stems and is the primary component of bacterial cell walls. During elongation in *A*. *tumefaciens*, nascent PG insertion is constrained to the pole. Polar growth is a characteristic of Rhizobiales and does not require the canonical MreB-RodA-PBP2 elongation complex. Indeed, all members of Rhizobiales have lost this complex entirely [14,15]

We showed that PBP1a is essential in *A*. *tumefaciens* and is the primary driver of polar growth. Depletion of PBP1a eliminates nascent PG insertion at the growth pole, leading to shorter cells that have compositional changes in PG [16]. In addition to its role in polar PG insertion, here we observe that during PBP1a depletion cells spread apart rather than form microcolonies. To better understand this phenotype, we used RNA-seq to obtain transcriptional profiles of cells depleted of PBP1a after 6 hours, corresponding to the onset of the spreading phenotype, and after 16 hours. Transcriptomic changes closely mimic the transcriptome changes seen when ChvG-ChvI is activated in *A*. *tumefaciens*, including downregulation of genes for motility and chemotaxis and upregulation of genes for exopolysaccharide biosynthesis and T6SS. Here we experimentally validate the RNA-seq results, confirming the impacts of PBP1a depletion on the physiology and behavior of *A*. *tumefaciens*.

## Results

### PBP1a depletion prevents proper microcolony formation

**Fig 1:**
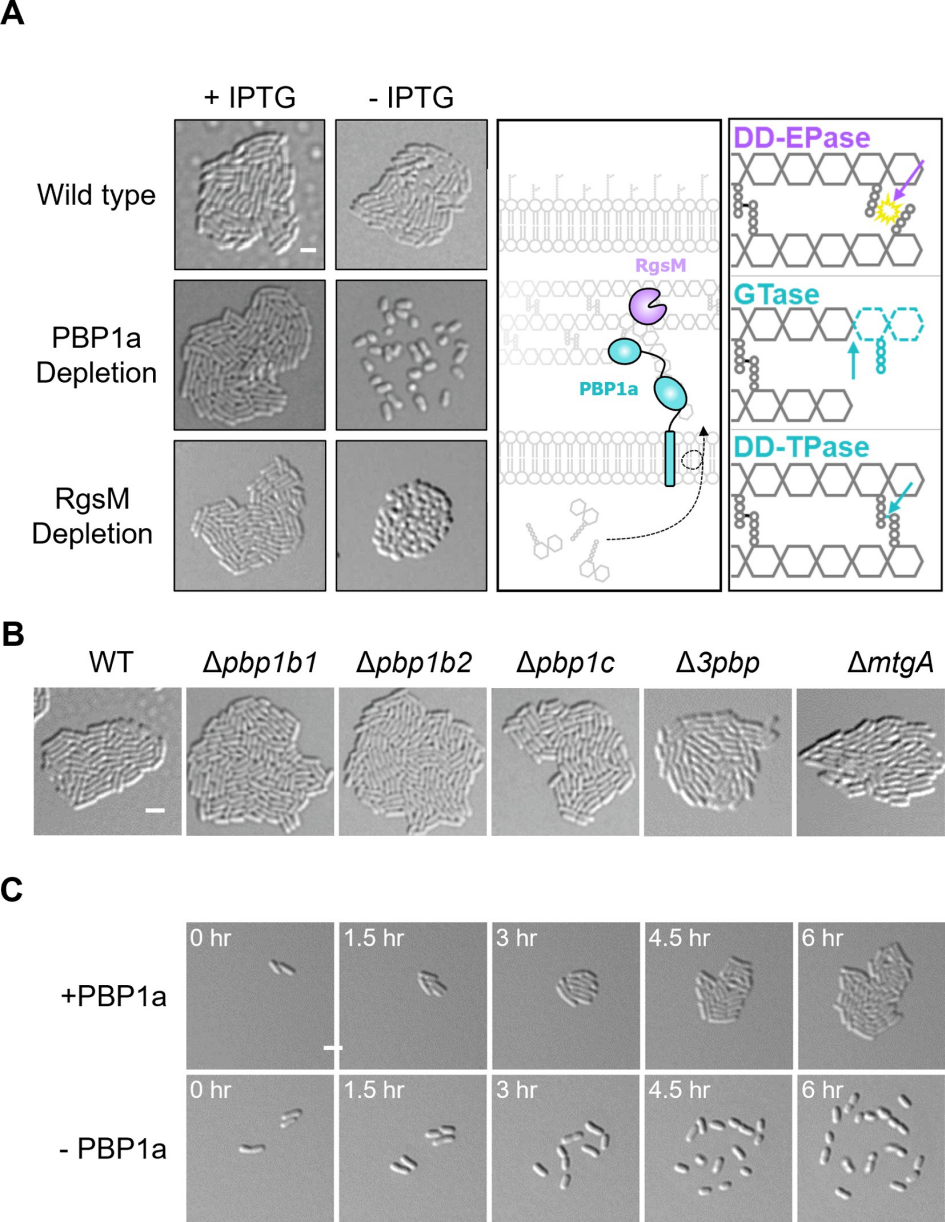
The PBP1a depletion fails to form microcolonies independent of flagellar motility. A. Micrographs of wildtype, PBP1a depletion, and RgsM depletion with or without 1mM IPTG inducer. Each strain was grown to exponential phase, spotted on an ATGN agar pad, allowed to grow for 16 hours, and imaged by DIC microscopy. Scale bar depicts 2μm. The graphic depicts the working model that RgsM _DD_-endopeptidase activity is required for incorporation of nascent glycan strands into the preexisting peptidoglycan (PG) macromolecule by PBP1a. RgsM cleaves _DD_-crosslinks, PBP1a glycosyltransferase activity incorporates lipid II into the PG glycan strand, PBP1a _DD_-transpeptidase activity crosslinks the peptide stem of the nascent PG, fully incorporating it into the macromolecule. EPase, endopeptidase; GTase, glycosyltransferase; TPase, transpeptidase. B. Micrographs of wild type, Δ*pbp1b1*, Δ*pbp1b2*, Δ*pbp1c*, and Δ*mtgA*. Each strain was grown to exponential phase, spotted on an ATGN agar pad, allowed to grow for 16 hours, and imaged by DIC microscopy. Scale bar depicts 2μm. C. Time-lapse microscopy of the PBP1a depletion grown on an agar pad with or without 1mM IPTG inducer. DIC images were acquired every 10 minutes. Time is shown in hours. For the—PBP1a strain, cells were washed 3X with ATGN media and grown at 28 C with shaking for 4 hours before cells were spotted on an agar pad for imaging.

Here, we grew PBP1a depleted cells on agarose pads and saw that these cells exhibit surface spreading rather than forming closely packed microcolonies (Fig 1A). Additionally, when centrifuging cultures of PBP1a-depleted cells, we observed that the cells did not pellet (S1 Fig). Considering the possibility that depletion of PBP1a somehow signals for these phenomena, we decided to look at RgsM, another enzyme required for polar elongation. Previous work points to RgsM activity being required for incorporation of nascent PG by PBP1a [17]. However, depletion of RgsM did not cause surface spreading (Fig 1A) indicating that an imbalance of PG hydrolysis and synthesis triggers spreading and the inability pellet in *A*. *tumefaciens*. Deletions of genes encoding other high molecular weight PBPs and *mtgA*, a PG transglycosylase, did not induce spreading (Fig 1B).

Timelapse microscopy revealed that after ~6 hours of PBP1a depletion cells spread apart, though the movement of cell appears to be confined within a relatively small region of the agarose pad (Fig 1C and S1 Movie). Since spreading is confined and occurs over the course of many hours, we suspected that this phenomenon was not simply caused by the activation of swimming motility.

### PBP1a depletion induces global transcriptome changes

**Fig 2:**
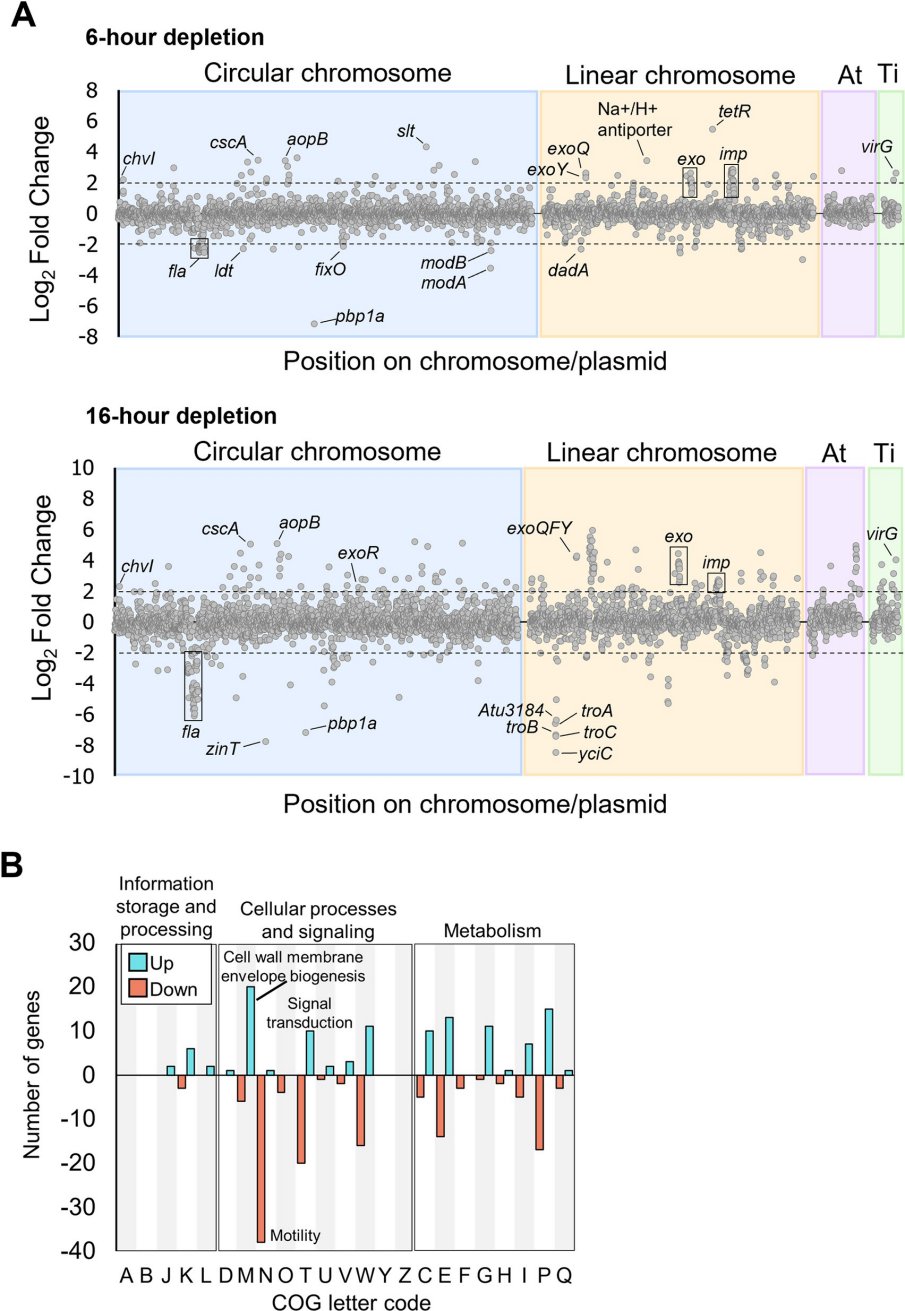
Analysis of the PBP1a depletion transcriptomes by RNA-seq. A. Plots comparing Log2Fold Change of the + PBP1a transcriptome to that of the—PBP1a 6-hour transcriptome and to that of the 16-hour depletion. Gray dots represent a single transcript, and the dotted lines represent +/- 2.0 Log2Fold Change threshold. Plots are delimited by chromosome and mega plasmid. B. COG categorical analysis of the 16-hour depletion of PBP1a. Pink, downregulated; Cyan, upregulated.

To understand the spreading phenotype caused by PBP1a depletion, we compared the transcriptomes of cells at the onset or late stage of the surface spreading phenotype. Cells were grown with or without the inducer Isopropyl β-D-1-thiogalactopyranoside (IPTG) for *mrcA*, encoding PBP1a, expression for 6 or 16 hours (Fig 2A). As a baseline, we compared transcriptional profiles of WT in the presence and absence of IPTG to the PBP1a depletion strain in the presence of IPTG. The addition of IPTG did not alter gene expression profiles of WT cells, and only minor differences were apparent between the PBP1a depletion strain background and WT when both strains are grown in the presence of IPTG (S2 Fig). We next compared differences in the PBP1a replete strain to the PBP1a depleted strain at either 6- or 16-hours post depletion (Fig 2A). Using a false discovery rate of < 0.05 and log2 fold-change (L2FC) > 2.0, we identified 91 and 306 genes that were differentially expressed in the + PBP1a strain compared to the 6- or 16-hour depletion, corresponding to 2% and 6% of the total genes, respectively.

Overall, we observed large-scale changes in a diverse and widespread range of genes that are regulated in response to PBP1a depletion. Initially, the response to PBP1a depletion is primarily mediated by chromosomally encoded rather than plasmid encoded genes. *A*. *tumefaciens* has a circular chromosome, which houses roughly half (51.7%) of the protein-coding genes, a linear chromosome (34.7%) and two mega plasmids, the At plasmid (10%) and Ti plasmid (3.6%). Most of the genes differentially expressed at both time points during PBP1a depletion were encoded on the linear and circular chromosomes (Fig 2A). Most of the differentially abundant genes from the 6-hour timepoint were also present in the 16-hour timepoint. For several of these genes, the magnitude of differential transcript abundance remained relatively constant. For example, the response regulator ChvI, had an increased relative abundance early in response to PBP1a depletion that remained constant in the 16-hour timepoint. In contrast, several genes displayed a continuous increase or decrease in transcript abundance between the 6- and 16-hour timepoints, including genes that encode proteins necessary for assembly of flagella and type 6 secretion system machinery. Finally, several genes were only differentially abundant at the 16-hour timepoint, including many genes encoding proteins important for cell envelope homeostasis such as the Tol-Pal system [18], and >30 ABC transporters.

To further categorize the diverse set of differentially abundant genes we identified Clusters of Orthologous Groups (COGs) in the 16-hour timepoint and classified them based on functional categories represented by a single letter code (Fig 2B) [19,20]. The most affected COG category was motility (N). Decreased abundance of mRNAs containing genes which encode structural flagella proteins further supports the hypothesis that the spreading phenotype is independent of flagella-based motility. The COG category with the largest proportion of increased differentially abundant genes was cell wall, membrane, and envelope biogenesis (M). Notably, no significant changes in the transcripts of other penicillin-binding proteins or glycosyltransferases were observed in response to loss of PBP1a (S1 Table). However, significant changes in transcripts encoding cell wall remodeling enzymes such as LD-transpeptidases, endopeptidases, and soluble lytic transglycosylases were detected (S1 Table). Atu0844, an LD-transpeptidase, was strongly downregulated suggesting it may play an important role in polar growth alongside PBP1a. Additionally, one putative β-lactamase gene, Atu0933, was strongly upregulated, which may provide a strategy to protect against cell wall damage. In addition, many of the genes found in this COG category encode cell envelope homeostasis and osmotic stress response proteins, including the Tol-Pal system, several outer membrane proteins (i.e. AopB), and periplasmic sensors (i.e. CreD).

At the 16-hour timepoint, the largest changes to cellular metabolism occurred in the inorganic ion transport and metabolism (P) and amino acid metabolism and transport (E) COG categories, suggesting a shift in nutrient uptake and metabolism. These changes resemble genes under control of RpoH1 in *Sinorhizobium meliloti*, which regulates expression of genes encoding ABC transporters, cell wall biosynthetic machinery, and membrane biogenesis proteins [21]. Furthermore, the 16-hour depletion of PBP1a shows a notable upregulation in transcription of Atu2445, encoding an RpoH ortholog (L2FC = 0.66), a stark increase from the 6-hour depletion (L2FC = 0.04) suggesting that the sigma factor RpoH may contribute to the observed changes in associated with nutrient uptake and metabolism during cell wall stress.

Interestingly, the six most downregulated genes in the 16-hour timepoint, with the exception of *mrcA*, encoding PBP1a, were *yciC* (Atu3181), *zinT* (Atu1049), *troC* (Atu3180), *troB* (Atu3179), *troA* (Atu3179), and Atu3184, all of which are major components of cytoplasmic zinc uptake in *A*. *tumefaciens* (Fig 2A, bottom) [22].

We also observed large increases and decreases in the transcript abundance of signal transduction genes. Transcription of *exoR* (Atu1715), *chvG* (Atu0033), and *chvI* (Atu0034) were upregulated at both the 6-hour (L2FC = 0.995, 1.42, and 2.18) and 16-hour (L2FC = 2.20, 1.41, and 2.27) depletions of PBP1a. Transcription of genes encoding additional signalling systems were also upregulated (S3 Fig and S1 Table).

### Transcriptome changes during PBP1a depletion mimic activation of the ChvG-ChvI two-component system

**Fig 3:**
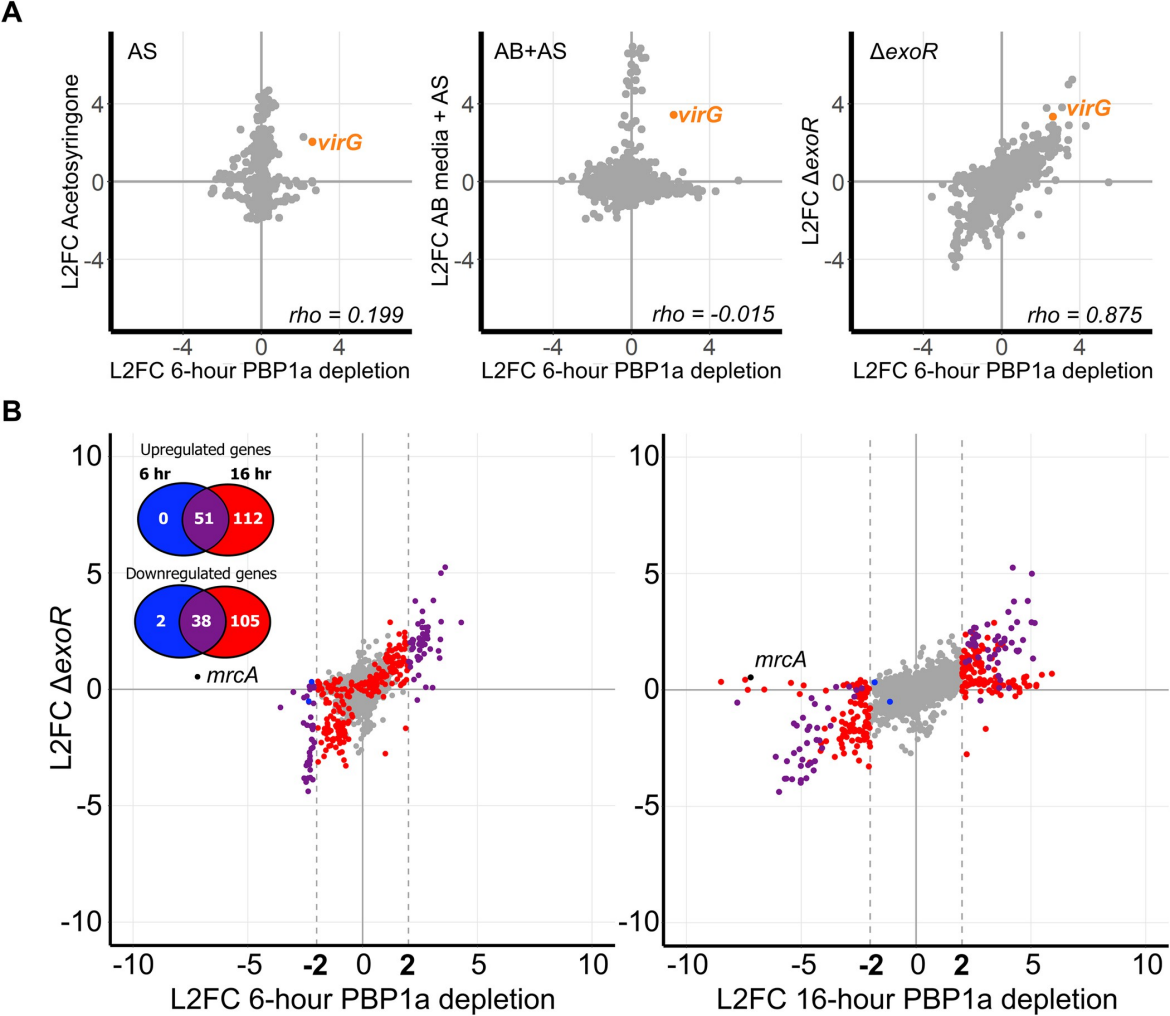
The response to the depletion of PBP1a mimics transcriptional changes associated with host invasion. A. Correlation scatterplots depicting relationships between the log2fold-change (L2FC) values in the 6-hour PBP1a depletion and transcriptomic data sets taken under simulated virulence-inducing conditions (AS and AB+AS) and under simulated host-invading conditions (Δ*exoR*). Each point represents a unique transcript. AS, acetosyrinogone; AB, *Agrobacterium* minimal media; rho, Spearman correlation coefficient. B. Correlation scatterplots comparing L2FC values of transcripts in the Δ*exoR* microarray to either the 6-hour or 16-hour PBP1a depletion. Each transcript is colored according to its change in L2FC values from 6 hours of PBP1a depletion to 16 hours of depletion. Gray, no change; Blue, transcript has |L2FC| > 2.0 in the 6-hour but not in the 16-hour depletion; Red, |L2FC| > 2.0 in the 16-hour but not in the 6-hour depletion; Purple, |L2FC| > 2.0 in both the 6-hour and 16-hour depletion.

Transcription of *virG*, encoding a TCS response regulator, was also strongly upregulated in both the 6-hour (L2FC = 2.61) and 16-hour (L2FC = 4.02) timepoints (S3 Fig and S1 Table). Transcription of *virG* has been reported to be upregulated under both host-invasion and virulence-inducing conditions [11]. Because *virG* was also upregulated during depletion of PBP1a, we reasoned that PBP1a depletion may be mimicking one of these two conditions. Using comparative transcriptomics, we compared the 150 most differentially expressed genes (DEGs) against published datasets that simulate host-invasion conditions (Δ*exoR* & pH 5.5) and virulence-inducing conditions (acetosyringone treatment & growth on AB media) [23,24]. We found that L2FC values of the 6-hour PBP1a depletion RNA-seq dataset correlated with the two host-invasion conditions and not with the virulence-inducing datasets, as indicated by the spearmen rho correlation coefficient for each comparison (Δ*exoR*, rho = 0.875; pH 5.5, rho = 0.766) (Figs 3A and S4). Rho values near 1 indicate similar DEGs between each dataset. Rho values near 0 would indicate no similar DEGs between each dataset (Fig 3A). Interestingly, each of these two datasets have been implicated in activation of the ChvG-ChvI pathway [9,11]. Correlation with each strongly implicates ChvG-ChvI activation in our RNA-seq dataset, suggesting that depletion of PBP1a may provide a signal leading to changes similar to those described to occur during the transition to a host-invasion lifestyle. While this trend was maintained in the 16-hour timepoint, we observed additional genes that were differentially expressed under depletion of PBP1a, but not in the Δ*exoR* and pH 5.5 datasets (Figs 3A and S4B). Indeed, the rho values for the 16-hour depletion of PBP1a compared to the host-invasion datasets (Δ*exoR*, rho = 0.529; pH 5.5, rho = 0.739 were lower than the 6-hour comparisons. Additionally, we found 215 more genes with L2FC > 2.0 in the 16-hour depletion than in the 6-hour depletion (Fig 3B). Together, these findings suggest that longer depletions of PBP1a may result in the activation of additional regulons beyond ChvG-ChvI.

Overall, a large number and variety of genes are regulated in response to depletion of PBP1a. Although many of these changes in gene expression have been reported previously in response to low pH or deletion of the ChvG-ChvI negative regulator ExoR, these changes have never been associated with loss of a cell wall synthase in *A*. *tumefaciens*. These observations indicate that there are additional mechanisms that can activate the ChvG-ChvI TCS.

### Succinoglycan overproduction is required for cell spreading

**Fig 4:**
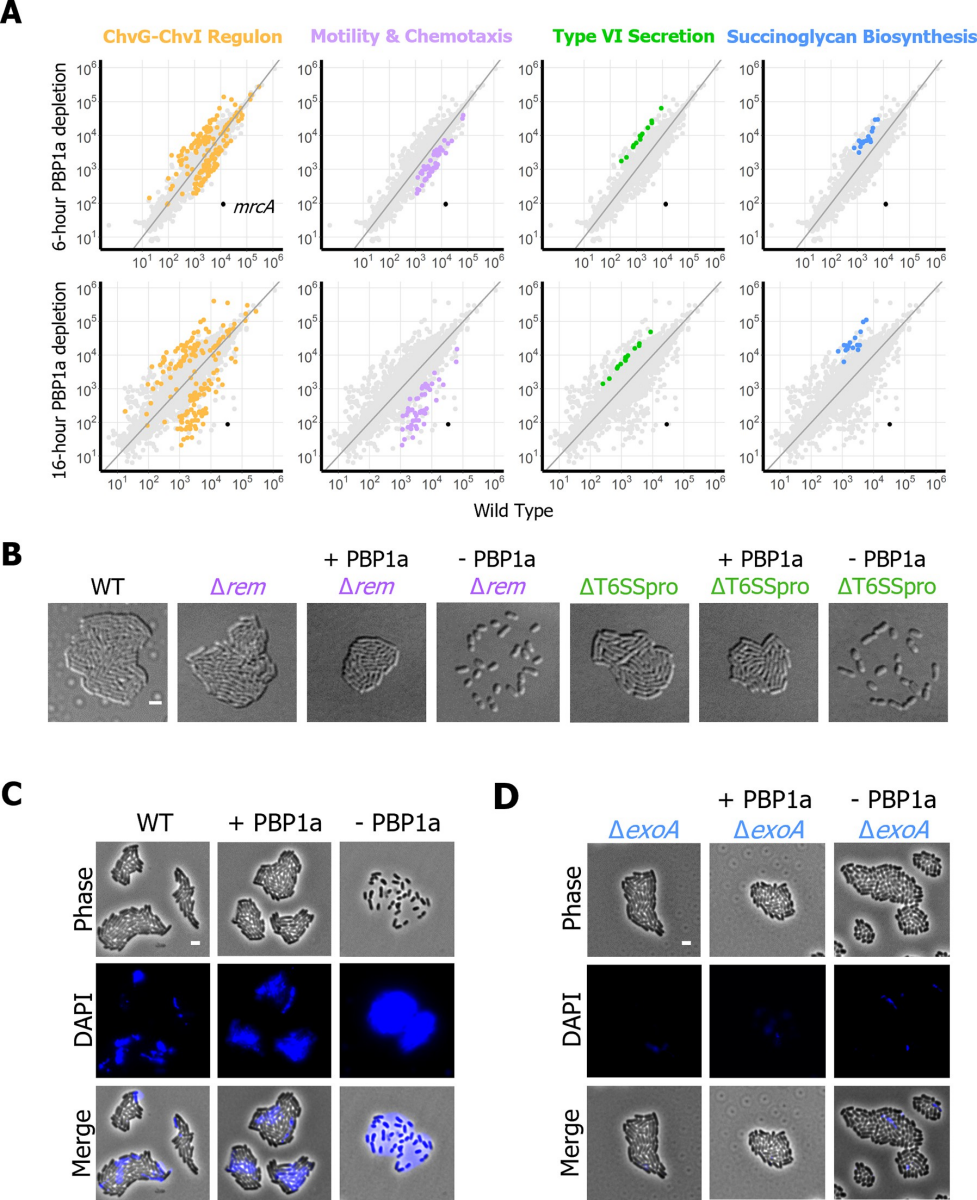
Succinoglycan overproduction is a conserved response to PBP1a depletion and results in failed microcolony formation. A. Scatter plots depicting RPKM values of the 6-hour and 16-hour compared to wild type. Each point represents a unique transcript. Points are colored by category. Gold, ChvG-ChvI regulon; Lavender, Motility and Chemotaxis; Green, Type VI Secretion; Blue, Succinoglycan Biosynthesis; Black, *mrcA* (encoding PBP1a). B. Micrographs of wild type, Δ*rem*, PBP1a replete Δ*rem*, PBP1a depleted Δ*rem*, ΔT6SSpro, PBP1a replete ΔT6SSpro, and PBP1a depleted ΔT6SSpro. Each strain was grown to exponential phase, spotted on a 1% ATGN agar pad containing 1mM IPTG if inducing *mrcA*, allowed to grow for 16 hours, and imaged by DIC microscopy. The scale bar depicts 2μm. C. Micrographs of wild type, and PBP1a depletion with or without IPTG inducer. Each strain was grown to exponential phase and spotted on a 1% ATGN agar pad containing 25μg/mL calcofluor white and 1mM IPTG if inducing *mrcA*. Each was allowed to grow for 16 hours and imaged by phase microscopy with and without the DAPI filter for visualizing calcofluor-stained succinoglycan. D. Micrographs of Δ*exoA* and PBP1a depletion Δ*exoA*, with or without IPTG inducer. Strains were grown and imaged as described for panel C.

Previous work has clearly associated activation of ChvG-ChvI to a specific transcriptomic pattern involving downregulation of flagellar motility genes and upregulation of T6SS and succinoglycan biosynthesis genes [9,11,25]. Indeed, this same pattern was observed in the 6- and 16-hour PBP1a depletion datasets (Fig 4A). To confirm that the spreading phenotype is unrelated to flagella-dependent motility, we made an in-frame deletion of *rem*, which encodes a transcriptional regulator of genes encoding structural flagella proteins [26,27], in the PBP1a depletion strain. Deletion of *rem* prevents swimming in *A*. *tumefaciens* and does not impact microcolony formation on agarose pads (Fig 4B). Upon depletion of PBP1a, *rem* mutants continued to spread, suggesting that the cause of this phenotype is independent of flagella-mediated swimming motility (Fig 4B).

All genes in the *imp* and *hcp* operons, which are located on the linear chromosome and encode the structural and toxin proteins of T6SS respectively, are upregulated at both timepoints (Fig 4A). In *A*. *tumefaciens*, activation of the T6SS results in the production of a contractile nanomachine which delivers effector proteins to antagonize and compete with other bacteria [28]. Among agrobacteria, T6SS is activated by different signals, is important during different stages of the lifecycle, and may be used to acquire nutrients [29]. To determine if the T6SS contributes to the spreading response observed during PBP1a depletion we deleted the intergenic gap between the *hcp* and *imp* operons (ΔT6SSpro). This deletion prevented expression of proteins from both the *hcp* and *imp* operons (S5 Fig) [30]. Cell spreading persisted in ΔT6SSpro during depletion of PBP1a suggesting that the activation of T6SS is not responsible for this behavior (Fig 4B).

Another possibility is that spreading might be caused by sliding motility, where secretion of a surfactant gives the cells a slippery surface to “slide” across. Notably, *S*. *meliloti* has been reported to undergo entropy-driven surface spreading during the overproduction of succinoglycan [31]. Succinoglycan is a β-1,4-linked sugar made of glucose and galactose, and is the most abundant exopolysaccharide produced by *A*. *tumefaciens* and related bacteria [32]. Genes associated with the biosynthesis and secretion of succinoglycan were strongly upregulated in both timepoints. To test if entropy-driven surface spreading is causing PBP1a-depleted *A*. *tumefaciens* cells to spread, we used a microscopy-based assay to observe succinoglycan production in *A*. *tumefaciens*. Cells were spotted on agarose pads containing calcofluor white and grown overnight, then imaged using the DAPI filter to detect succinoglycan production (Fig 4C). Wild-type *A*. *tumefaciens* and the PBP1a replete strains secrete some succinoglycan that enriched near the center of the microcolony (Fig 4C). In comparison, depletion of PBP1a triggers secretion of a large quantity of succinoglycan that defines the boundary of where the cells spread. An in-frame deletion of *exoA*, which encodes a glycosyltransferase required for succinoglycan production in *A*. *tumefaciens* [33], prevents succinoglycan production (Fig 4D). During PBP1a depletion, microcolony formation is restored in the Δ*exoA* mutant (Fig 4D). Together, these data illustrate that succinoglycan overproduction contributes to the surface spreading of the PBP1a depletion.

### Deletion of *chvG* or *chvI* results in hypersensitivity to β-lactam antibiotics

Since activation of succinoglycan production is known to be part of the ChvG-ChvI regulon, we next wanted to test if the PBP1a depletion is activating SGN production through the ChvG-ChvI signaling pathway. We made an in-frame deletion of *chvI* in the PBP1a depletion background and found *chvI* mutants replete with PBP1a appear morphologically wild-type when grown in minimal media (S6A Fig). However, PBP1a-depleted Δ*chvI* cells were extremely sick. Previously we reported that the PBP1a depletion produces viable daughter cells for up to 5–6 generations [16], however, the PBP1a-depleted Δ*chvI* strain was incapable of a single division event. Instead, the cells exhibited growth arrest and cell lysis shortly after depletion initiation (S6A Fig and S2 Movie).

**Fig 5:**
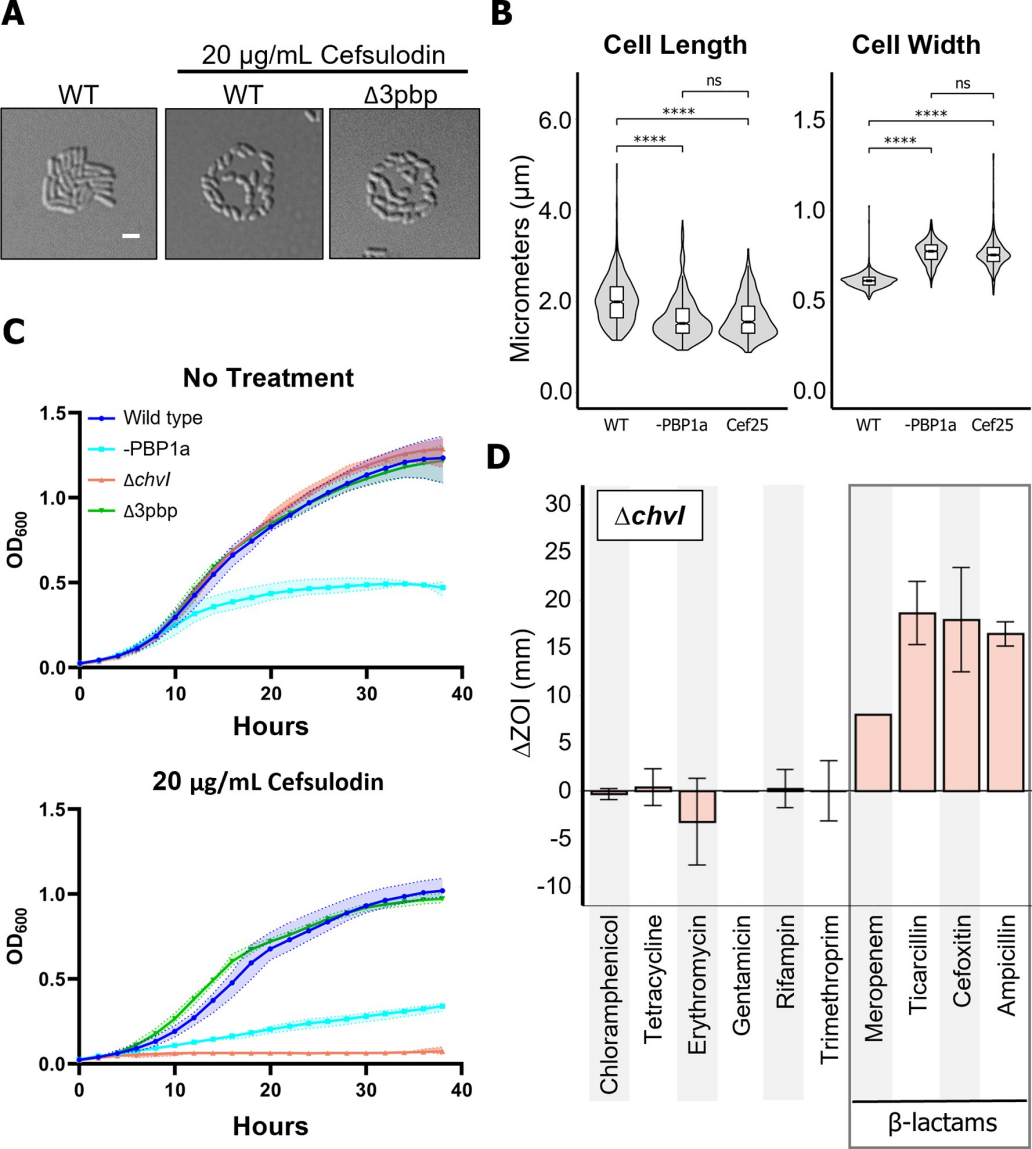
The ChvG-ChvI TCS is conditionally essential under treatment with β-lactam antibiotics. A. Micrographs of untreated and cefsulodin-treated cells. Wild-type and Δ3pbp cells were grown to exponential phase, spotted on a 1% ATGN agar pad with or without 20 μg/mL cefsulodin and allowed to grow for 16 hours. Each strain was imaged by DIC microscopy. B. Box plots comparing cell length and width between wild-type, PBP1a-depleted, and cefsulodin-treated cells. ns, not significant; ****, p < 0.00005. C. Growth curves of WT, -PBP1a, Δ*chvI*, and Δ*3pbp* in the absence (top) and presence of 20 μg/mL cefsulodin (bottom). D. Graph depicting the change in zone of inhibition from wildtype in Δ*chvI* against ten different antibiotic disks. Error bars represent +/- 1 standard deviation from the mean.

To further assess the enhanced sensitivity of Δ*chvI* to PBP1a depletion, we identified an antibiotic that likely targets PBP1a enzymatic activity. Treatment with cefsulodin at a concentration of 20 μg/mL resulted in a cell spreading phenotype similar to the PBP1a depletion (Fig 5A). The presence of cefsulodin induces spreading in the triple PBP mutant (Δ3*pbp*) which lacks all of the high molecular weight PBPs other than PBP1a [16] suggesting that targeting of PBP1a is sufficient to cause this phenotype (Fig 5A). Treatment with cefsulodin results in short, round cells, phenocopying the PBP1a depletion (Fig 5B). In addition, the Δ3*pbp* mutant does not have obvious resistance to cefsulodin as would be expected if a primary drug target was absent (Figs 5C and S6C). Together, these data suggest that cefsulodin targets the transpeptidase activity of PBP1a in *Agrobacterium tumefaciens*, similar to reported cefsulodin specificity in *E*. *coli* [34]. Next, we observed relative cefsulodin sensitivities in WT, Δ*chvI*, and Δ*exoR* strains. Remarkably treatment with 10 μg/mL of cefsulodin deceases viability of Δ*chvG* and Δ*chvI* cells by 5 orders of magnitude compared to either WT or Δ*exoR* cells (S6B Fig). Furthermore, growth curve analysis reveals that Δ*chvI* cells are unable to grow within an hour of exposure to cefsulodin at concentrations that are sublethal for WT cells (Figs 5C and S6C). Overall, these findings suggest that the ChvG-ChvI TCS is essential for growth when the activity of the major PG synthase is inhibited either chemically or genetically.

We broadened our investigation by testing Δ*chvG* and Δ*chvI* against ten additional antibiotics: four that block protein synthesis (chloramphenicol, tetracycline, erythromycin, and gentamicin); one that blocks DNA replication (nalidixic acid); one that blocks transcription (rifampin); and four other cell wall synthesis inhibiting β-lactam antibiotics (meropenem, cefoxitin, ampicillin, and ticarcillin). To measure changes in sensitivity to each antibiotic compared to wildtype, Δ*chvG* and Δ*chvI* were spread on ATGN minimal media and disks containing each antibiotic were applied. Diameters of the zones of inhibition (ZOI) were measured and the difference in ZOIs for each mutant strain compared to wildtype are shown (Figs 5D and S6D). Of the antibiotics tested, Δ*chvG* and Δ*chvI* showed increased sensitivity only to β-lactam antibiotics, suggesting specificity of the ChvG-ChvI pathway in conferring resistance to this antibiotic class.

### ChvG and ChvI are conserved in Alphaproteobacteria but the presence of ExoR is more constrained

**Fig 6:**
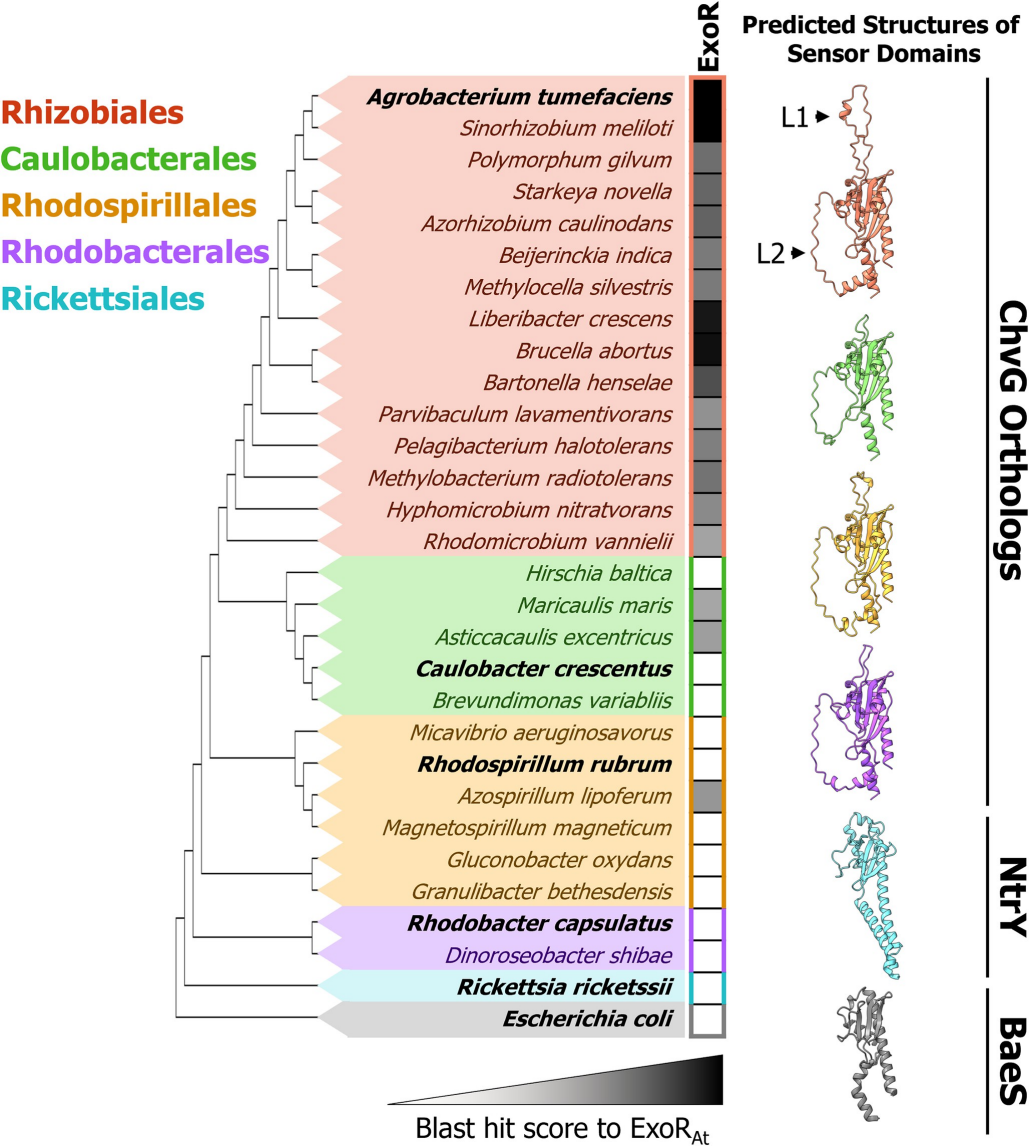
Conservation constraints of ExoR suggest conserved ChvG-ChvI response is independent of ExoR. Maximum parsimony tree constructed using MUSCLE sequence alignment [67] on the periplasmic regions of ChvG orthologs. In clades that don’t have a ChvG ortholog, the protein with the highest sequence similarity to ChvG was used instead. Conservation of ExoR was calculated using blast max scores from top hits when protein blasting [66] ExoR from *Agrobacterium tumefaciens* against each species in the tree. Phyre2 [71] predicted structures of periplasmic domains of ChvG orthologs from representatives (bold) in each genus are shown. Conserved structural loops are denoted as L1 and L2.

**Fig 7:**
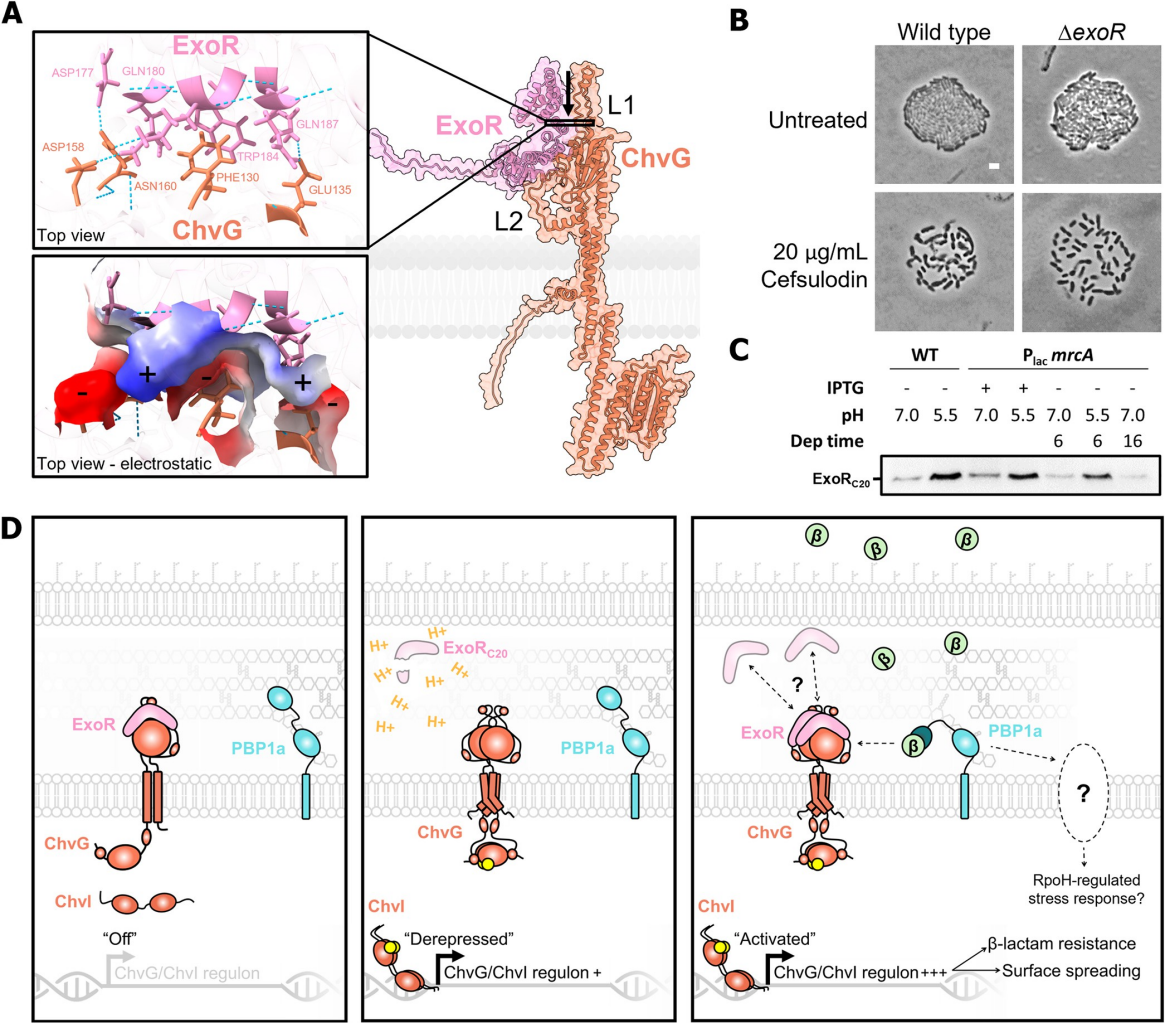
Activation of ChvG-ChvI can proceed independently of ExoR derepression. A. Predicted interaction site between ExoR and ChvG through AlphaFold-Multimer structure prediction. Insets show the top-down view of the interaction site with hydrogen bonding and electrostatic surface display. B. Microcolonies of wild type and Δ*exoR* with and without 20 μg/mL cefsulodin treatment. C. Western blot detection of ExoR proteolysis (ExoR_C20_) with anti-FLAG against ExoR-FLAG. D. Working model of activation of ChvG-ChvI in *A*. *tumefaciens*. H^+^, free proton representing an acidic environment; β, β-lactam antibiotic.

The absence of PBP1a activity at the growth pole during elongation activates ChvG-ChvI, the canonical host-invasion pathway of *Agrobacterium tumefaciens*. The ChvG-ChvI pathway is most well known to be activated by environmental changes associated with conditions favorable for plant association, yet this TCS is retained in many non-plant-associated Alphaproteobacteria (Fig 6). Remarkably, while ChvG-ChvI is conserved in a large proportion of Alphaproteobacteria, ExoR is not (Fig 6) [6]. Predicted structures of the sensor domains of ChvG in bacteria with ChvG-ChvI orthologs show two structural loops (L1 and L2; Figs 6 and S7 and S8). While L2 is conserved across the orthologous structures, L1 is expanded solely in the Rhizobiales (S8 Fig). This expansion coincides with the retention of ExoR, making it a compelling target for ExoR-ChvG association studies (Fig 6). Structural loops are often sites of protein-protein interaction and can be vital to protein function [35]. To explore the possibility that these loops may play a role in the docking of ExoR we used AlphaFold-Multimer to detect the interaction interface between ExoR and ChvG [36]. Indeed, an interaction site in L1 comprised of hydrogen bonding and an electrostatic pocket was revealed (Fig 7A). This may indicate that ExoR*-*dependent repression of ChvG is dependent on L1. Chen et al. identified suppressor mutants in *S*. *meliloti* to a ChvI mutant displaying decreased activity (S9 Fig) [37]. Two suppressors were substitutions that disrupted ExoR interaction with ChvG (also known as ExoS in *S*. *meliloti*), G76C and S156Y. S156Y falls near the predicted interaction site between L1 and ExoR (S9 Fig). A computational study by Wiech et al. proposed three possible interaction interfaces between ChvG (ExoS) and ExoR, of which site B is in agreement with our putative interaction interface (S9 Fig) [38].

Either proteolysis of ExoR or deletion of *exoR* is sufficient to elicit transcriptional changes associated with activation of ChvG-ChvI, including the upregulation in genes associated with succinoglycan biosynthesis [6,7,11,39]. We reasoned that if ChvG-ChvI activation initiates surface spreading, we should be able to detect the spreading in an *exoR* mutant. While Δ*exoR* microcolonies appear slightly less crowded than wild type microcolonies, they do not phenocopy the PBP1a depletion nor treatment with cefsulodin (Fig 7B). Remarkably, cell spreading is induced by the Δ*exoR* mutant by the presence of cefsulodin (Fig 7B). To monitor ExoR proteolysis we introduced a C-terminal FLAG-tagged copy of ExoR into the wild type and PBP1a depletion strains (S10 Fig). While elevated levels of the ExoR cleavage product (ExoR_C20_) were readily detected by acid-induction (pH 5.5), depletion of PBP1a for 6 hours and 16 hours in neutral conditions (pH 7.0) does not result in accumulation of ExoR_C20_ (Figs 7C and S10C). These data are consistent with the phenotypic characterization of Δ*exoR* and together these results suggest that cell wall stress activates the ChvG-ChvI two-component system independently of ExoR.

We hypothesized that the ChvG-ChvI pathway may confer resistance to cell wall stress in other host-associated Rhizobiales. Indeed, depletion of PBP1a in *Sinorhizobium meliloti* causes cells to spread suggest activation of ChvG-ChvI (ExoS-ChvI) pathway (S11A Fig). Like *A*. *tumefaciens*, this response is specific to depletion of PBP1a and is not triggered by deletion of the 5 other high molecular weight PBPs. These results suggest that cell wall stress may be a well conserved trigger for activation of ChvG-ChvI pathways in the Rhizobiales. Notably, *C*. *crescentus* does not spread upon treatment with 80 μg/mL of cefsulodin (S11B Fig), despite recent findings that ChvGI likely confers resistance at this concentration [12]. While *S*. *meliloti* and *A*. *tumefaciens* encode succinoglycan biosynthesis operons, *C*. *crescentus* does not. These findings support the hypothesis that the ChvG-ChvI response to cell wall stress is conserved across Alphaproteobacterial species that have orthologs of ChvG-ChvI.

## Discussion

Why is ChvG-ChvI TCS activated upon inhibition of polar PG synthesis in *A*. *tumefaciens*? PBP1a depletion results in a compositional shift in cell wall muropeptide composition and cell wall crosslinking [16]. Here we find that PBP1a depletion causes upregulation in transcription of LD-transpeptidases, endopeptidases, and soluble lytic transglycosylase, indicating cells are attempting to compensate for compromised cells walls. Remarkably, transcripts from these same genes are upregulated in the Δ*exoR* and pH 5.5 datasets, suggesting that remodeling of the cell wall is a part of the ChvG-ChvI regulon. While the role of cell wall remodeling during host-invasion is unclear it is possible that these modifications may be protective for the bacterium when host associated. The upregulation in transcription of genes encoding AopB and the Tol/Pal system may indicate that PBP1a-depleted cells are succumbing to osmotic pressure, a possible explanation for the increase in cell width in PBP1a-depleted and cefsulodin-treated cells (Fig 5B). The increased sensitivity to mecillinam, vancomycin, cefsulodin, and moenomycin in *C*. *crescentus* ChvGI mutants [12] along with our observation that ChvG-ChvI mutants in *A*. *tumefaciens* are hypersensitive to β-lactam antibiotics suggests that this pathway may confer resistance to antibiotics inhibiting cell wall synthesis. This aligns well with previous studies in *S*. *meliloti* demonstrating that *chvG-chvI* mutants have a unique lipopolysaccharide profile [40] and increased sensitivity to detergents [41]. In addition, the *Brucella abortus* ChvG-ChvI orthologs (BvrS-BvrR) initiate extensive transcriptional changes associated with the outer membrane [42–44]. Together, these findings indicate a conserved role within the Alphaproteobacteria for the ChvG-ChvI TCS in sensing and responding to envelope stress. Possible signals may include the accumulation of cytoplasmic peptidoglycan precursors such as lipid II, increased levels of naked glycan strands in the cell wall, or more conventional stress responses due to osmotic sensitivity [45]. Notably, Gieger et al. found that a phospholipid mutant of *S*. *meliloti* activates ChvG-ChvI (ExoS-ChvI) in an ExoR-independent manner [46]. The authors proposed that absence of phosphatidylcholine, a major membrane phospholipid causes a conformational change in ChvG, initiating autophosphorylation. It is tempting to consider a similar type of activation during depletion of PBP1a, but our observation that the depletion of *A*. *tumefaciens* RgsM does not induce surface spreading (Fig 1A) hints that increased cell wall hydrolysis may be responsible for activation of the ChvG-ChvI pathway. While our findings suggest a conserved signal in ChvG-ChvI activation, further studies will need to be conducted to identify the signal(s), determine if they are species-specific, and explore the conditions which lead to signal production.

If there is a conserved role in sensing cell wall stress, why would this pathway be required for host invasion within *Agrobacterium tumefaciens*? One explanation could be that during plant colonization, *A*. *tumefaciens* decreases cell wall biogenesis to form cells which are relatively persistent in order to evade host recognition and survive the harsh *in plantae* environment [47]. Alternatively, perhaps the integrity and composition of the cell wall is routinely monitored and used as a signal for the activation of pathways associated with lifestyle choice. Decreased activity of PBP1a may mimic one or more of the conditions *A*. *tumefaciens* and *S*. *meliloti* encounter during host-invasion, leading to the activation of ChvG-ChvI. Another possibility is that the absence of PBP1a activity leads to a destabilization of the polar growth complex leading to decreased cell envelope integrity. It will be of interest to determine if other components of the polar growth complex such as GPR [48], RgsP [49], or PopZ [50,51] have increased sensitivity to β-lactam antibiotics and induce surface spreading. This possibility is in agreement with the observation in *C*. *crescentus* that resistance to cell wall targeting antibiotics is dependent on factors such as TipN that maintain the integrity of the cell envelope [13].

The overproduction of succinoglycan provides cells with passive protection against several stresses *A*. *tumefaciens* may encounter during host invasion including detergents, salt, acidity, heat, antimicrobial peptides, and reactive oxygen species [32,33]. Production of succinoglycan may also help protect against cell-wall-synthesis targeting antibiotics produced by competing bacteria and fungi in the soil. However, succinoglycan production is taxonomically constrained within plant-host-associated bacteria, indicating that there are other conserved mechanisms regulated by ChvG-ChvI in resistance to these cell envelope stressors. The surface spreading phenomenon that we connected to overproduction of succinoglycan and that has been previously described in *S*. *meliloti* [31], may be involved in cell dispersal during host invasion.

The role of ExoR regulation is well established for the acid-induction of ChvG-ChvI [9,11,25]. While deletion of *exoR* upregulates expression of succinoglycan biosynthesis genes [11], it is insufficient to initiate surface spreading. Three pieces of evidence indicate that activation of ChvG-ChvI during cell wall stress proceeds independently of ExoR. First, ExoR conservation is a characteristic of Rhizobiales, but the ChvG-ChvI two-component system is more broadly conserved in Alphaproteobacteria. Second, Δ*exoR* is not more resistant to cefsulodin than wild type. Finally, proteolysis of ExoR occurs in low pH conditions, but not during depletion of PBP1a. One possibility is that derepression of ChvG alone is not sufficient and an alternative mechanism of ChvG activation results in a more robust response. Cell wall stress (ie PBP1a depletion, β-lactam treatment) may cause a signal for ChvG activation to accumulate. Alternatively, ChvI binding to DNA may be enhanced by an unknown mechanism under cell wall stress conditions. The observation that both *ΔchvG and ΔchvI* are hypersensitive to cefsulodin (S6B Fig) and other β-lactam antibiotics (Figs 5D and S6C) suggests that ChvG plays an important role in sensing and responding to cell wall stress and not that ChvI is activated through an alternative histidine kinase. The observation that sublethal concentrations of cefsulodin result in a complete block in growth of Δ*chvI* cells (Fig 5C) indicates that ChvI is required for survival during cell wall stress through activation of the ChvGI regulon. Future experiments will be needed to identify the signals which accumulate during cell wall stress, determine if the signal is sensed directly or indirectly by ChvG, and how activation of ChvI is achieved.

Together our findings support a model in which ChvG-ChvI is repressed by ExoR (Fig 7D, left) but can be derepressed by acid through dissociation and proteolysis of ExoR in a Rhizobiales-specific response (Fig 7D, middle). We posit that ChvG-ChvI is activated through a more broadly conserved cell envelope stress response (Fig 7D, right). Lastly, longer periods of PBP1a depletion result in a more general stress response beyond the ChvG-ChvI regulon (Fig 7D, right), which may be mediated by Atu2445, an ortholog of sigma factor RpoH.

Overall, the data presented here are in agreement with recent works in *Caulobacter* [12,13] which suggest that ChvGI activation is important in oligotrophic free-living bacteria as a cell envelope or osmotic stress response. Remarkably, it seems that the ChvG-ChvI pathway has a dual purpose in protecting the bacterium and invading its host in *A*. *tumefaciens*, and other host-associated Rhizobiales.

## Materials and methods

### Bacterial strains, plasmids, and growth conditions

A list of all bacterial strains and plasmids used in this study is provided in S2 Table. *Agrobacterium tumefaciens* C58 and derived strains were grown in ATGN minimal media [52] without exogenous iron at 28°C with shaking. When appropriate, kanamycin (KAN) was used at the working concentration of 300 μg/ml. When indicated, isopropyl β-D-1-thio-galactopyranoside (IPTG) was used as an inducer at a concentration of 1 mM. *Sinorhizobium meliloti* stains were grown in TY medium [53] at 28°C. When appropriate, KAN was used at the working concentration of 200 μg/ml, gentamycin (GM) was used at 20 μg/ml, and IPTG was used at a concentration of 2 mM. *C*. *crescentus* strains were grown in PYE medium [54] at 28°C. *E*. *coli* DH5α and S17-1 λ pir were grown in Lysogeny Broth medium at 37°C and when appropriate 50 μg/ml or 30 μg/ml of KAN were added, respectively.

### Construction of plasmids and strains

A list of all primers and synthetic DNAs used in this study is provided in S3 Table. Vectors for gene deletion by allelic exchange were constructed using recommended methods for *A*. *tumefaciens* [55]. Briefly, 500-bp fragments upstream and 500 bp downstream of the target gene were amplified using primer pairs P1/P2 and P3/P4 respectively. Amplicons were spliced together by SOEing using primer pair P1/P4. The amplicon was digested and ligated into pNTPS139. The deletion plasmids were introduced into *A*. *tumefaciens* by mating using an *E*. *coli* S17 conjugation strain to create kanamycin resistant, sucrose sensitive primary integrants. Primary integrants were grown overnight in media with no selection. Secondary recombinants were screened by patching for sucrose resistance and kanamycin sensitivity. Colony PCR with primers P5/P6 for the respective gene target was used to confirm deletion. PCR products from P5/P6 primer sets were sequenced to further confirm deletions.

The ExoR-FLAG expression vector was constructed by amplifying *exoR* without a stop codon using *exoR* Forward and Reverse primers. The PCR amplicon was digested and ligated into pFLGC-2, generating pFLGC-2*exoR-flag*, containing a copy of *exoR* with an in-frame c-terminal flag tag behind a vanillate-inducible promoter which functions constitutively in *A*. *tumefaciens* [56]. pFLGC-2*exoR-flag* was sequenced using Plasmidsaurus. pFLGC-2*exoR-flag* was introduced into *A*. *tumefaciens* by mating using the S17 conjugation strain to create kanamycin resistant colonies. Transformation was verified by colony PCR using pVMCS Forward and Reverse primers.

### Phase and fluorescence microscopy

A small volume (~1 μl) of cells in exponential phase (OD600 = 0.2–0.4) was applied to a 1% ATGN agarose pad as described previously [57]. DIC, phase contrast and epifluorescence microscopy were performed with an inverted Nikon Eclipse TiE and a QImaging Rolera em-c2 123 1K EMCCD camera with Nikon Elements Imaging Software. For time-lapse microscopy, images were collected every ten minutes, unless otherwise stated. For calcofluor agar pad assays, calcofluor was added to agarose pads at a concentration of 25 μg/mL and exposed to DAPI filter for 50 ms. When appropriate agar pads were supplemented with 1mM IPTG. For quantitative image analysis, live cells were imaged using phase-contrast microscopy, and cell length and width distributions of the indicated number of cells per strain were determined as measured using MicrobeJ software [58]. T-tests were performed using the compare_means() function of the ggpubr R library.

### RNA isolation, sequencing and analysis

Four cultures each of WT, WT + IPTG and 12 cultures of + PBP1a depletion cells were grown overnight in 2 ml of ATGN minimal media at 28°C with shaking; the + PBP1a strains and WT + IPTG strains were supplemented with 1mM IPTG. Cells were then pelleted by centrifugation at 7000 x g for 5 minutes. Cell pellets were washed three times with ATGN by centrifugation and resuspension to remove IPTG. After the final wash the cell pellets from WT, WT + IPTG, and four of the 12 + PBP1a strains were resuspended to an OD600 of 0.05 in 6mL ATGN, or ATGN with 1mM IPTG. The other eight + PBP1a strains were resuspended to an OD600 of 0.05 in 6 ml ATGN without IPTG. This resulted in 4 replicate cultures each of WT, WT +IPTG, + PBP1a,—PBP1a_6hr and—PBP1a_16hr. Growth of the cultures was monitored and supplemented with fresh medium as needed so that the OD600 never went over 0.3. RNA was isolated from the -PBP1a_6hr strains after 6 hours of growth, and RNA was isolated from all other strains after 16 hours of growth. To prepare samples, a culture volume equivalent to 6 ml at an optical density at 600 nm (OD600) of 0.2–0.3 was pelleted by centrifugation at 7000 x g for 5 minutes and pellets were resuspended in 1mL of ATGN media and incubated with 2 mL of RNAProtect reagent (QIAgen) for 15 min at room temperature. Cells were lysed with 10 mg lysozyme, and RNA was extracted using the QIAgen RNEasy kit.

DNA libraries for sequencing were constructed following the manufacturer’s protocol with reagents supplied in Illumina’s TruSeq mRNA stranded sample preparation kit without the steps to enrich for poly-A mRNA. The sample concentration was determined by Qubit flourometer (Invitrogen) using the Qubit HS RNA assay kit, and the RNA integrity was checked using the Fragment Analyzer automated electrophoresis system. Briefly, RNA is fragmented, double-stranded cDNA is generated from fragmented RNA, and the index containing adapters are ligated to the ends. The amplified cDNA constructs were purified by addition of Axyprep Mag PCR Clean-up beads. The final construct of each purified library was evaluated using the Fragment Analyzer automated electrophoresis system, quantified with the Qubit flourometer using the Qubit HS dsDNA assay kit, and diluted according to Illumina’s standard sequencing protocol for sequencing on the NextSeq 500.

For all samples, when adapter sequence was detected, it was removed using cutadapt (0.16) [59]. All samples were purged of reads that mapped to transcripts for rRNA genes using bowtie2 (2.3.4.3) [60]. The reads were then mapped to the *A*. *fabrum* str. C58 genome using STAR (version 2.5.4b) [61], which also produces the number of read counts per gene. The index files used by STAR were derived from the files *Agrobacterium*_fabrum_str_c58.ASM9202v1.dna.toplevel.fa and *Agrobacterium*_fabrum_str_c58.ASM9202v1.40.gtf, both of which are part of Ensembl release 40 (http://bacteria.ensembl.org/index.html). Pairwise comparisons were performed to test for differential expression of genes using the Bioconductor package DESeq2 [62]. Gene annotations were collected from the annotations included with the file of cDNAs also at Ensembl *Agrobacterium*_fabrum_str_c58.ASM9202v1.cdna.all.fa.gz.

### COG functional annotation

Amino acid sequences for all proteins in *A*. *tumefaciens* were downloaded in a single FASTA file from GenBank and uploaded to EGGNOG-MAPPER [63,64]. COG terms were outputted, and Python code was written to pull out transcripts from 16-hour depletion of PBP1a with L2FC > 2.0 or < -2.0. Some transcripts had multiple COG annotations and were therefore replicated for visualization according to the number of annotations it had.

### Comparative transcriptomics

Transcripts and L2FC values from each dataset were opened in Python code written to screen for and exclude any genes that were not present in both datasets. Statistics and visualization was done in R. Spearman correlation statistical test was run on the L2FC of the 150 most differentially expressed genes in the PBP1a depletion and their corresponding L2FC values in the comparison dataset.

### Cell viability assays

For cell viability spot assays, cultures were grown overnight and diluted to an OD_600_ = 0.05 and serially diluted in ATGN and spotted on ATGN agar plates containing antibiotics as indicated. Four microliters of each dilution was spotted and plates were incubated at 28°C for 48 h before imaging.

### Disk diffusion assays

Wild-type, Δ*chvG*, and Δ*chvI* cells were overnight and then knocked down to an OD_600_ of 1.0. Cells were then lawned on ATGN minimal media. Sterile paper disks either soaked in concentrations of each antibiotic or not (blank controls) were applied to the plate. Each plate was grown for ~48 hours at 28°C before being imaged. Zone of inhibition diameters were measured from each image using ImageJ software.

### Phylogenetics and structure prediction

A seed of 22 amino acid sequences containing the annotated ChvG sensor domain (PF13755) were initially downloaded from Pfam [65]. Each was blasted against its corresponding proteome to retrieve the full protein sequence [66]. Additional sequences of relevant bacteria such as *S*. *meliloti*, *Brucella melitensis*, and *C*. *crescentus*, were added by blasting the amino acid sequence from *A*. *tumefaciens* ChvG (Atu0033) against each organism’s proteome. All sequences were aligned using MUSCLE and trimmed in Jalview according to Uniprot predicted periplasmic region of Atu0033 [67–69]. A maximum parsimony phylogenetic tree of these sequences was generated using MEGA-X [70].

Each trimmed sequence underwent one-to-one threading in Phyre2 with the complete structure of Atu0033 predicted by AlphaFold as a template [71,72]. Local alignment and a secondary structure weight of 0.1 was used. Structural analysis and structure alignment was done in ChimeraX [73].

The amino acid sequence of *A*. *tumefaciens* ExoR (Atu1715) was blasted against each organism’s proteome and max score values of top hits were recorded. Max score values under 50 were deemed too different and were therefore not considered an ExoR ortholog. Additionally, sequences of each top hit were blasted against the proteome of *A*. *tumefaciens*. If the top hit was not ExoR, it was also not considered an ExoR ortholog in this analysis.

ExoR-ChvG interaction predictions were made in AlphaFold-Multimer through the Google Colab service [36]. Hydrogen-bonding and electrostatic predictions were made using the ChimeraX software.

### Western blot analysis

Two cultures of wild type were grown overnight in 1 mL ATGN each supplemented with 300 μg/mL kanamycin and six cultures of PBP1a depletion were grown in 1 mL ATGN each supplemented with 300 μg/mL kanamycin and 1mM IPTG. Overnight cells were knocked down to an OD600 of 0.1 and allowed to grow in fresh media supplemented with kanamycin and if necessary IPTG for four hours. Cells were pelleted and washed 3 times. One WT pellet and three PBP1a depletion pellets were resuspended in 25 mL ATGN supplemented with 300 μg/mL kanamycin. One of these resuspended PBP1a pellets was also supplemented with 1mM IPTG. The other WT pellet and three PBP1a pellets were resuspended in 25 mL of ATGN buffered to pH 5.5 with 200 mM MES supplemented with 300 μg/mL kanamycin. One of these resuspended PBP1a pellets was also supplemented with 1mM IPTG. All resuspensions were placed in a shaking incubator at 28°C. WT and +PBP1a cultures were removed when OD600 reached 0.3–0.4. The 6-hour PBP1a depletion cultures were monitored for 6 hours, and fresh media was added so that they never rose above an OD600 of 0.4. The 16-hour depletions were monitored for 16 hours with fresh media added so that they never rose above 0.4. No growth was detected in the 16-hour depletion grown at pH 5.5 and this culture was not processed further. All cultures were pelleted at 5000 x g for 15 minutes and resuspended in 1 mL of Qiagen B1 Lysis Buffer and 100 ug/mL of lysozyme was added to each sample. The samples were vortexed at max speed and incubated for 30 minutes at 37°C.

Protein concentrations were measured using a Pierce BCA Protein Assay Kit. Each sample was normalized to 1 μg/mL of protein. 10 μL of 4X loading buffer was added to 30 μL of each sample. All nine protein samples with 4X loading buffer were boiled for five minutes and added to a 4–20% Bis-Tris GenScript *SurePAGE* gel. BlueStain Protein ladder (P007-500) was loaded into the first well. Proteins were transferred to a PVDF membrane cut to the size of the gel using a BioRad Thermo-Blot-Turbo-Transfer device. The membrane was blocked for 1 hour in 20 mL of 5% milk in TBS + 0.05% Tween 20. 1:1000 dilution of HRP-conjμgated Anti-DYKDDDDK mouse monoclonal antibody (Invitrogen) was added, and the membrane was gently shaken overnight at 4°C. The membrane was washed 3 times with fresh TBS + 0.05% Tween 20 for 5 minutes each. Immediately after wash steps, membranes were transferred to TBS + 0.05% Tween 20 with 1:10000 dilution of goat anti-mouse IgG secondary antibody (Invitrogen) and gently shaken for 1 hour. The membrane was washed 3 times with fresh TBS + 0.05% Tween 20 and then developed for 5 minutes using SuperSignal West Femto Maximum Sensitivity Substrate (34095). The membrane was imaged using a BioRad ChemiDoc Imager.

## Supporting information

S1 Movie Growth and morphological changes during 8 hours of PBP1a depletion. Cells were washed to remove inducer and spotted immediately on an ATGN pad. Images were acquired every ten minutes and movie is played at 16 frames per second for a total of 48 frames.
(MP4)

S2 Movie Growth and division of Δ*chvI* during PBP1a depletion. Cells were washed to remove inducer and spotted immediately on an ATGN pad. Images were acquired every five minutes and movie is played at 40 frames per second for a total of 200 frames.
(MP4)

S1 FigPelleting of PBP1a-depleted and repleted cells.Conical tubes show turbidity after pelleting cells grown for 16 hours in PBP1a replete (+PBP1a) or depleted (-PBP1a) conditions. Cells were centrifuged at 1690 x g (3000 rpm in TX-400 rotor in a Sorvall Legend X1R centrifuge) for 10 minutes. Supernatants were spotted on a 1.25% ATGN agarose pad. $\bar{x}$ = average number of cells from 10 fields of view.(TIF)

S2 Fig Analysis of the control transcriptomes by RNA-seq. A. Plots comparing Log2Fold Change of the WT 6-hour transcriptome to that of the WT +ITPG 6-hour transcriptome. Gray dots represent a single transcript, and the dotted lines represent +/- 2.0 Log2Fold Change threshold. Plots are delimited by chromosomes and mega plasmids. B. Plots comparing Log2Fold Change of the WT +IPTG transcriptome to that of the PBP1a depletion strain with ITPG present to drive PBP1a expression. Comparisons shown are of the 6-hour transcriptomes. Gray dots represent a single transcript, and the dotted lines represent +/- 2.0 Log2Fold Change threshold. Plots are delimited by chromosomes and mega plasmids.
(TIF)

S3 Fig Transcriptional changes of TCS regulators and kinases during PBP1a depletion. The fold change in expression level of TCS regulators and kinases are shown following 6 hours (gray) and 16 hours (black) of PBP1a depletion. The *virAG* and *chvGI* TCS pairs are labeled.
(TIF)

S4 Fig The response to the depletion of PBP1a mimics transcriptional changes associated with host invasion. A. Correlation scatterplots depicting relationships between the log2fold-change (L2FC) values in the 16-hour PBP1a depletion and transcriptomic data sets taken under simulated virulence-inducing conditions (AS) and under simulated host-invading conditions (Δ*exoR*). Each point represents a unique transcript. AS, acetosyrinogone; Rho, Spearman correlation coefficient. B. Correlation scatterplots comparing L2FC values of transcripts in the pH 5.5 microarray, a condition known to induce the *chvG-chvI* regulon, to either the 6-hour (red) or 16-hour (blue) PBP1a depletion. Rho, Spearman correlation coefficient.
(TIF)

S5 Fig Western blot of proteins expressed from the two type VI secretion system operons in ΔT6SSpro strains. Top panel, diagram of the two operons encoding elements of Type VI Secretion in *A*. *tumefaciens*. T6SSpro labels the intergenic gap that is deleted in ΔT6SSpro strains. Middle panel, western blots using anti-Hcp and anti-TssB in each of the indicated strains. Protein sizes (kDa) are shown on the right. Bottom panel, Coomassie stained gel showing total protein from each strain. Western blots were performed as described in the methods with the following modifications. Lysates were prepared by pelleting cells via centrifugation and resuspending in 1X loading buffer. Next, the suspension was run through a 20G needle for lysis. 3 gels were loaded with identical concentrations of sample. BlueStain2 Protein ladder (P008-500) was loaded into the first well of each. One of the three gels was Coomassie stained at room temperature with gentle shaking for 10–15 minutes and imaged for total protein content. The other two gels were transferred to immobilon-FL transfer membranes, blocked with 0.5% milk, and transferred to a solution of TBS + 0.05% Tween 20 with 4 μL of 1:1000 dilution of either anti-TssB or anti-Hcp for 1 hour. Membranes were washed and transferred to TBS + 0.05% Tween 20 with 4 μL of 1:1000 dilution of anti-rabbit HRP goat IgG for 1 hour.
(TIF)

S6 Fig Impact of decreased PG synthesis on *A*. *tumefaciens*. A. Micrographs of PBP1a depletion Δ*chvI* with (+PBP1A) or without (-PBP1A) IPTG. Cells were grown to exponential phase in ATGN media containing IPTG, spotted on an ATGN agarose pad with or without IPTG, allowed to grow for 16 hours, and imaged by DIC microscopy. B. Cell viability of each wild type, Δ*chvG*, Δ*chvI*, and Δ*exoR* spotted on an ATGN agar plate containing 10 μg/mL of cefsulodin. Ten-fold serial dilutions are indicated. C. Growth curves of WT, -PBP1a, Δ*chvI*, and Δ*exoR*, Δpbp3, Δ*mtgA* in the absence (black line) and presence of 20 μg/mL cefsulodin (colored line). D. Graph depicting the change in zone of inhibition from wildtype in Δ*chvG* against ten different antibiotic disks. Error bars represent +/- 1 standard deviation from the mean.
(TIF)

S7 Fig Alignment of periplasmic regions of ChvG orthologs. Partial MUSCLE alignment of ChvG ortholog periplasmic domains. Highlighted columns represent strong conservation across aligned sequences. Atu0033 (ChvG of *A*. *tumefaciens)* is the reference sequence for this analysis. L1 and L2 correspond to two conserved structural loops. Conservation, quality, and consensus scores for each site are represented as bar graphs under the alignment. Shading indicates Order of the bacterium containing the ChvG ortholog: Orange, Rhizobiales; Purple, Rhodobacterales; Green, Caulobacterales; Gold, Rhodospirales.
(TIF)

S8 Fig Structure predictions for the periplasmic regions of ChvG orthologs. Phyre2 structural predictions for each organism displayed in Fig 6A of this work. Genus and species names as well as locus tags for each ChvG ortholog are provided. Range of numbers following the back slash are the amino acid sites used in structure prediction. Colors indicates order of the bacterium containing the ChvG ortholog: Orange, Rhizobiales; Purple, Rhodobacterales; Green, Caulobacterales; Gold, Rhodospirales.
(TIF)

S9 Fig Comparisons of putative interaction sites between ExoR and ChvG. Previously characterized suppressor mutants (Chen et al. 2008) or predicted interaction sites (Wiech et al. 2014) are mapped onto the ExoR-ChvG AlphaFold Multimer structure presented here. The full structure is shown at the top left with detailed views of the ExoR-ChvG interface shown as the protein complex is rotated (front, side, and back).
(TIF)

S10 Fig ExoR-FLAG proteolysis. A. Schematic of P_van_ driven expression of a ExoR-FLAG fusion protein. The sequence of the FLAG tag is shown above. The plasmid was introduced in the WT and PBP1a depletion strains to enable monitoring of ExoR proteolysis. B. Potential ExoR products are shown, including the pro-ExoR (ExoR_P_), mature ExoR (ExoR_M_), and cleaved ExoR (ExoR_C20_). ExoR_P_ and ExoR_M_ were indistinguishable and therefore denoted as ExoR. C. Western blot demonstrating ExoR-FLAG cleavage following acid treatment but not PBP1a depletion. Bands corresponding to ExoR_C20_ are labeled. Resolution of the ExoR on the blot does not allow for distinguishing between ExoR_P_ and ExoR_M_.
(TIF)

S11 Fig Surface spreading is taxonomically constrained to succinoglycan producing bacteria. A. Micrographs of *Sinorhizobium meliloti* Rm2011 PBP1a replete, PBP1a depleted, and a strain with deletions of genes encoding all other high molecular weight PBPs (Δ5pbp). Each strain was grown to exponential phase, spotted on a 1% TY agar pad containing 1mM IPTG if inducing *mrcA*, allowed to grow for 16 hours, and imaged with phase microscopy. B. Micrographs of WT *C*. *crescentus* cells growth with or without cefsulodin. Cells were grown to exponential phase in PYE media, spotted on a PYE agar pad with or without cefsulodin, allowed to grow for 16 hours, and imaged by DIC microscopy. All scale bars depict 2 μm.
(TIF)

S1 Table Selected differentially expressed genes. Genes related to cell wall synthesis and remodeling, cell envelope homeostasis, and signalling are shown following 16 hours of PBP1a depletion. Two-component signalling systems are shaded together. The degree of differential regulation is indicated by shading in the log_2_FC column: red indicates downregulation, white indicates no significant change, and teal indicates upregulation.
(XLSX)

S2 Table Bacterial strains and plasmids. Descriptions of bacterial strains and plasmids used in this study. References and sources are provided when relevant.
(XLSX)

S3 Table Synthesized DNA primers. The sequences of primers used to construct plasmids and strains in this study are provided. All primers were ordered from IDT.
(XLSX)

## References

[1] Venturi V, Keel C. Signaling in the rhizosphere. Trends Plant Sci. 2016;21: 187–198. doi: 10.1016/j.tplants.2016.01.005 26832945

[2] Capra EJ, Laub MT. Evolution of two-component signal transduction systems. Annu Rev Microbiol. 2012;66: 325–347. doi: 10.1146/annurev-micro-092611-150039 22746333 PMC4097194

[3] Yuan Z-C, Haudecoeur E, Faure D, Kerr KF, Nester EW. Comparative transcriptome analysis of *Agrobacterium tumefaciens* in response to plant signal salicylic acid, indole-3-acetic acid and γ-amino butyric acid reveals signalling cross-talk and *Agrobacterium* -plant co-evolution. Cell Microbiol. 2008;10: 2339–2354. doi: 10.1111/j.1462-5822.2008.01215.x 18671824

[4] Cho H, Winans SC. VirA and VirG activate the Ti plasmid *repABC* operon, elevating plasmid copy number in response to wound-released chemical signals. Proceedings of the National Academy of Sciences. 2005;102: 14843–14848. doi: 10.1073/pnas.0503458102 16195384 PMC1253548

[5] Xue S, Biondi EG. Coordination of symbiosis and cell cycle functions in *Sinorhizobium meliloti*. Biochimica et Biophysica Acta (BBA)—Gene Regulatory Mechanisms. 2019;1862: 691–696. doi: 10.1016/j.bbagrm.2018.05.003 29783033

[6] Heavner ME, Qiu W-G, Cheng H-P. Phylogenetic co-occurrence of ExoR, ExoS, and ChvI, components of the RSI bacterial invasion switch, suggests a key adaptive mechanism regulating the transition between free-living and host-invading phases in Rhizobiales. Mergaert P, editor. PLoS One. 2015;10: e0135655. doi: 10.1371/journal.pone.0135655 26309130 PMC4550343

[7] Wells DH, Chen EJ, Fisher RF, Long SR. ExoR is genetically coupled to the ExoS-ChvI two-component system and located in the periplasm of *Sinorhizobium meliloti*. Mol Microbiol. 2007;64: 647–664. doi: 10.1111/j.1365-2958.2007.05680.x 17462014

[8] Lu H-Y, Luo L, Yang M-H, Cheng H-P. *Sinorhizobium meliloti* ExoR is the target of periplasmic proteolysis. J Bacteriol. 2012;194: 4029–4040. doi: 10.1128/JB.00313-12 22636773 PMC3416547

[9] Yuan ZC, Liu P, Saenkham P, Kerr K, Nester EW. Transcriptome profiling and functional analysis of *Agrobacterium tumefaciens* reveals a general conserved response to acidic conditions (pH 5.5) and a complex acid-mediated signaling involved in Agrobacterium-plant interactions. J Bacteriol. 2008;190. doi: 10.1128/JB.01387-07 17993523 PMC2223696

[10] Alakavuklar MA, Heckel BC, Stoner AM, Stembel JA, Fuqua C. Motility control through an anti-activation mechanism in *Agrobacterium tumefaciens*. Mol Microbiol. 2021;116: 1281–1297. doi: 10.1111/mmi.14823 34581467 PMC8690355

[11] Heckel BC, Tomlinson AD, Morton ER, Choi JH, Fuqua C. *Agrobacterium tumefaciens* ExoR controls acid response genes and impacts exopolysaccharide synthesis, horizontal gene transfer, and virulence gene expression. J Bacteriol. 2014;196. doi: 10.1128/JB.01751-14 24982308 PMC4135700

[12] Quintero-Yanes A, Mayard A, Hallez R. The two-component system ChvGI maintains cell envelope homeostasis in *Caulobacter crescentus*. bioRxiv. 2022; 2022.01.18.476748. doi: 10.1101/2022.01.18.476748 PMC9731502 36480504

[13] Vallet SU, Hansen LH, Bistrup FC, Laursen SA, Chapalay JB, Chambon M, et al. Loss of bacterial cell pole stabilization in *Caulobacter crescentus* sensitizes to outer membrane stress and peptidoglycan-directed antibiotics. mBio. 2020;11. doi: 10.1128/mBio.00538-20 32371598 PMC7403779

[14] Brown PJB, de Pedro MA, Kysela DT, van der Henst C, Kim J, de Bolle X, et al. Polar growth in the Alphaproteobacterial Order Rhizobiales. Proc Natl Acad Sci U S A. 2012;109. doi: 10.1073/pnas.1114476109 22307633 PMC3277149

[15] Margolin W. Sculpting the bacterial cell. Current Biology. 2009. doi: 10.1016/j.cub.2009.06.033 19906583 PMC4080913

[16] Williams MA, Aliashkevich A, Krol E, Kuru E, Bouchier JM, Rittichier J, et al. Unipolar peptidoglycan synthesis in the Rhizobiales requires an essential class A penicillin-binding protein. mBio. 2021;12. doi: 10.1128/mBio.02346-21 34544272 PMC8546619

[17] Figueroa-Cuilan WM, Randich AM, Dunn CM, Santiago-Collazo G, Yowell A, Brown PJB. Diversification of LytM protein functions in polar elongation and cell division of *Agrobacterium tumefaciens*. Front Microbiol. 2021;12. doi: 10.3389/fmicb.2021.729307 34489918 PMC8416486

[18] Krol E, Yau HCL, Lechner M, Schäper S, Bange G, Vollmer W, et al. Tol-pal system and Rgs proteins interact to promote unipolar growth and cell division in *Sinorhizobium meliloti*. mBio. 2020;11. doi: 10.1128/MBIO.00306-20 32605980 PMC7327166

[19] Tatusov RL, Koonin VE, Lipman DJ. A genomic perspective on protein families. Science. 1997;278: 631–637. doi: 10.1126/science.278.5338.631 9381173

[20] Galperin MY, Kristensen DM, Makarova KS, Wolf YI, Koonin VE. Microbial genome analysis: the COG approach. Brief Bioinform. 2019;20: 1063. doi: 10.1093/bib/bbx117 28968633 PMC6781585

[21] de Lucena DK, Pühler A, Weidner S. The role of sigma factor RpoH1 in the pH stress response of *Sinorhizobium meliloti*. BMC Microbiol. 2010;10: 265. doi: 10.1186/1471-2180-10-265 20955556 PMC2976971

[22] Chaoprasid P, Dokpikul T, Johnrod J, Sirirakphaisarn S, Nookabkaew S, Sukchawalit R, et al. *Agrobacterium tumefaciens* Zur regulates the high-affinity zinc uptake system TroCBA and the putative metal chaperone YciC, along with ZinT and ZnuABC, for survival under zinc-limiting conditions. Appl Environ Microbiol. 2016;82: 3503. doi: 10.1128/AEM.00299-16 27060116 PMC4959167

[23] Yuan Z-C, Edlind MP, Liu P, Saenkham P, Banta LM, Wise AA, et al. The plant signal salicylic acid shuts down expression of the *vir* regulon and activates quormone-quenching genes in *Agrobacterium*. Proceedings of the National Academy of Sciences. 2007;104: 11790–11795. doi: 10.1073/pnas.0704866104 17606909 PMC1905925

[24] Haryono M, Cho S-T, Fang M-J, Chen A-P, Chou S-J, Lai E-M, et al. Differentiations in gene content and expression response to virulence induction between two *Agrobacterium* Strains. Front Microbiol. 2019;10: 1554. doi: 10.3389/fmicb.2019.01554 31354658 PMC6629968

[25] Wu C-F, Lin J-S, Shaw G-C, Lai E-M. Acid-induced type VI secretion system is regulated by ExoR-ChvG/ChvI signaling cascade in *Agrobacterium tumefaciens*. Ausubel FM, editor. PLoS Pathog. 2012;8: e1002938. doi: 10.1371/journal.ppat.1002938 23028331 PMC3460628

[26] Figueroa-Cuilan W, Daniel JJ, Howell M, Sulaiman A, Brown PJB. Mini-Tn7 insertion in an artificial *att*Tn7 site enables depletion of the essential master regulator *ctrA* in the phytopathogen *Agrobacterium tumefaciens*. Appl Environ Microbiol. 2016;82. doi: 10.1128/AEM.01392-16 27287320 PMC4968531

[27] Rotter C, Mühlbacher S, Salamon D, Schmitt R, Scharf B. Rem, a new transcriptional activator of motility and chemotaxis in *Sinorhizobium meliloti*. J Bacteriol. 2006;188: 6932–6942. doi: 10.1128/JB.01902-05 16980496 PMC1595514

[28] Wu C-F, Smith DA, Lai E-M, Chang JH. The *Agrobacterium* type VI secretion system: A contractile nanomachine for interbacterial competition. Current Topics in Microbiology and Immunology. Berlin, Heidelberg: Springer; 2018. pp. 215–231. doi: 10.1007/82_2018_99 29992360

[29] Wu C-F, Weisberg AJ, Davis EW, Chou L, Khan S, Lai E-M, et al. Diversification of the type VI secretion system in Agrobacteria. Comstock LE, editor. mBio. 2021;12: e01927–21. doi: 10.1128/mBio.01927-21 34517758 PMC8546570

[30] Lin J-S, Ma L-S, Lai E-M. Systematic dissection of the *Agrobacterium* type VI secretion system reveals machinery and secreted components for subcomplex formation. PLoS One. 2013;8: e67647. doi: 10.1371/journal.pone.0067647 23861778 PMC3702570

[31] Dilanji GE, Teplitski M, Hagen SJ. Entropy-driven motility of *Sinorhizobium meliloti* on a semi-solid surface. Proceedings of the Royal Society B: Biological Sciences. 2014;281: 20132575. doi: 10.1098/rspb.2013.2575 24741008 PMC4043075

[32] Mendis HC, Madzima TF, Queiroux C, Jones KM. Function of succinoglycan polysaccharide in *Sinorhizobium meliloti* host plant invasion depends on succinylation, not molecular weight. Winans SC, editor. mBio. 2016;7. doi: 10.1128/mBio.00606-16 27329751 PMC4916376

[33] Schmid J, Sieber V, Rehm B. Bacterial exopolysaccharides: biosynthesis pathways and engineering strategies. Front Microbiol. 2015;6: 496. doi: 10.3389/fmicb.2015.00496 26074894 PMC4443731

[34] Curtis NA, Orr D, Ross GW, Boulton MG. Affinities of penicillins and cephalosporins for the penicillin-binding proteins of *Escherichia coli* K-12 and their antibacterial activity. Antimicrob Agents Chemother. 1979;16: 533–539. doi: 10.1128/AAC.16.5.533 393164 PMC352901

[35] Espadaler J, Querol E, Aviles FX, Oliva B. Identification of function-associated loop motifs and application to protein function prediction. Bioinformatics. 2006;22: 2237–2243. doi: 10.1093/bioinformatics/btl382 16870939

[36] Evans R, O’Neill M, Pritzel A, Antropova N, Senior A, Green T, et al. Protein complex prediction with AlphaFold-Multimer. bioRxiv. 2022; 2021.10.04.463034. doi: 10.1101/2021.10.04.463034

[37] Chen EJ, Sabio EA, Long SR. The periplasmic regulator ExoR inhibits ExoS/ChvI two-component signalling in *Sinorhizobium meliloti*. Mol Microbiol. 2008;69: 1290–1303. doi: 10.1111/j.1365-2958.2008.06362.x 18631237 PMC2652646

[38] Wiech EM, Cheng H-P, Singh SM. Molecular modeling and computational analyses suggests that the *Sinorhizobium meliloti* periplasmic regulator protein ExoR adopts a superhelical fold and is controlled by a unique mechanism of proteolysis. Protein Science. 2015;24: 319–327. doi: 10.1002/pro.2616 25492513 PMC4353358

[39] Ratib NR, Sabio EY, Mendoza C, Barnett MJ, Clover SB, Ortega JA, et al. Genome-wide identification of genes directly regulated by ChvI and a consensus sequence for ChvI binding in *Sinorhizobium meliloti*. Mol Microbiol. 2018;110: 596–615. doi: 10.1111/mmi.14119 30192418 PMC6343485

[40] Wang C, Kemp J, da Fonseca IO, Equi RC, Sheng X, Charles TC, et al. *Sinorhizobium meliloti* 1021 loss-of-function deletion mutation in *chvI* and its phenotypic characteristics. Molecular Plant-Microbe Interactions®. 2010;23: 153–160. doi: 10.1094/MPMI-23-2-0153 20064059

[41] Charles TC, Nester EW. A chromosomally encoded two-component sensory transduction system is required for virulence of *Agrobacterium tumefaciens*. J Bacteriol. 1993;175: 6614–6625. doi: 10.1128/jb.175.20.6614–6625.1993 8407839 PMC206773

[42] Lamontagne J, Butler H, Chaves-Olarte E, Hunter J, Schirm M, Paquet C, et al. Extensive cell envelope modulation is associated with virulence in *Brucella abortus*. J Proteome Res. 2007;6: 1519–1529. doi: 10.1021/pr060636a 17343405

[43] Manterola L, Moriyón I, Moreno E, Sola-Landa A, Weiss DS, Koch MHJ, et al. The lipopolysaccharide of *Brucella abortus* BvrS/BvrR mutants contains lipid A modifications and has higher affinity for bactericidal cationic peptides. J Bacteriol. 2005;187: 5631–5639. doi: 10.1128/JB.187.16.5631–5639.2005 16077108 PMC1196083

[44] Viadas C, Rodríguez MC, Sangari FJ, Gorvel J-P, García-Lobo JM, López-Goñi I. Transcriptome analysis of the *Brucella abortus* BvrR/BvrS two-component regulatory system. Bereswill S, editor. PLoS One. 2010;5: e10216. doi: 10.1371/journal.pone.0010216 20422049 PMC2858072

[45] Piepenbreier H, Diehl A, Fritz G. Minimal exposure of lipid II cycle intermediates triggers cell wall antibiotic resistance. Nat Commun. 2019;10: 2733. doi: 10.1038/s41467-019-10673-4 31227716 PMC6588590

[46] Geiger O, Sohlenkamp C, Vera-Cruz D, Medeot DB, Martínez-Aguilar L, Sahonero-Canavesi DX, et al. ExoS/ChvI two-component signal-transduction system activated in the absence of bacterial phosphatidylcholine. Front Plant Sci. 2021;12. doi: 10.3389/fpls.2021.678976 34367203 PMC8343143

[47] Erbs G, Silipo A, Aslam S, de Castro C, Liparoti V, Flagiello A, et al. Peptidoglycan and muropeptides from pathogens *Agrobacterium* and *Xanthomonas* elicit plant innate immunity: Structure and activity. Chem Biol. 2008;15: 438–448. doi: 10.1016/j.chembiol.2008.03.017 18482696

[48] Zupan JR, Grangeon R, Robalino-Espinosa JS, Garnica N, Zambryski P. GROWTH POLE RING protein forms a 200-nm-diameter ring structure essential for polar growth and rod shape in *Agrobacterium tumefaciens*. Proc Natl Acad Sci U S A. 2019;166. doi: 10.1073/pnas.1905900116 31085632 PMC6561148

[49] Schäper S, Yau HCL, Krol E, Skotnicka D, Heimerl T, Gray J, et al. Seven-transmembrane receptor protein RgsP and cell wall-binding protein RgsM promote unipolar growth in Rhizobiales. Copenhaver GP, editor. PLoS Genet. 2018;14: e1007594. doi: 10.1371/journal.pgen.1007594 30102748 PMC6107284

[50] Howell M, Aliashkevich A, Salisbury AK, Cava F, Bowman GR, Brown PJB. Absence of the polar organizing protein PopZ results in reduced and asymmetric cell division in *Agrobacterium tumefaciens*. J Bacteriol. 2017;199. doi: 10.1128/JB.00101-17 28630123 PMC5553032

[51] Grangeon R, Zupan J, Jeon Y, Zambryski PC. Loss of PopZ At activity in *Agrobacterium tumefaciens* by deletion or depletion leads to multiple growth poles, minicells, and growth defects. mBio. 2017;8. doi: 10.1128/mBio.01881-17 PMC5686542 29138309

[52] Morton ER, Fuqua C. Laboratory Maintenance of *Agrobacterium*. Current Protocols in Microbiology. 2012. doi: 10.1002/9780471729259.mc03d01s24 22307549 PMC3350319

[53] BERINGER JE. R factor transfer in *Rhizobium leguminosarum*. Microbiology (N Y). 1974;84: 188–198. doi: 10.1099/00221287-84-1-188 4612098

[54] Poindexter JS. Biological properties and classification of the *Caulobacter* group. Bacteriol Rev. 1964;28: 231–295. doi: 10.1128/br.28.3.231-295.1964 14220656 PMC441226

[55] Morton ER, Fuqua C. Genetic manipulation of *Agrobacterium*. Curr Protoc Microbiol. 2012. doi: 10.1002/9780471729259.mc03d02s25 22549163 PMC3434950

[56] Thanbichler M, Iniesta AA, Shapiro L. A comprehensive set of plasmids for vanillate- and xylose-inducible gene expression in *Caulobacter crescentus*. Nucleic Acids Res. 2007;35: e137–e137. doi: 10.1093/nar/gkm818 17959646 PMC2175322

[57] Howell M, Daniel JJ, Brown PJB. Live cell fluorescence microscopy to observe essential processes during microbial cell growth. Journal of Visualized Experiments. 2017;2017. doi: 10.3791/56497 29286454 PMC5755469

[58] Ducret A, Quardokus EM, Brun Y v. MicrobeJ, a tool for high throughput bacterial cell detection and quantitative analysis. Nat Microbiol. 2016;1: 16077. doi: 10.1038/nmicrobiol.2016.77 27572972 PMC5010025

[59] Martin M. Cutadapt removes adapter sequences from high-throughput sequencing reads. EMBnet J. 2011;17: 10. doi: 10.14806/ej.17.1.200

[60] Langmead B, Salzberg SL. Fast gapped-read alignment with Bowtie 2. Nat Methods. 2012;9: 357–359. doi: 10.1038/nmeth.1923 22388286 PMC3322381

[61] Dobin A, Gingeras TR. Mapping RNA-seq Reads with STAR. Curr Protoc Bioinformatics. 2015;51: 11.14.1–11.14.19. doi: 10.1002/0471250953.bi1114s51 26334920 PMC4631051

[62] Love MI, Huber W, Anders S. Moderated estimation of fold change and dispersion for RNA-seq data with DESeq2. Genome Biol. 2014;15: 550. doi: 10.1186/s13059-014-0550-8 25516281 PMC4302049

[63] Sayers EW, Bolton EE, Brister JR, Canese K, Chan J, Comeau DC, et al. Database resources of the national center for biotechnology information. Nucleic Acids Res. 2022;50: D20–D26. doi: 10.1093/nar/gkab1112 34850941 PMC8728269

[64] Huerta-Cepas J, Szklarczyk D, Heller D, Hernández-Plaza A, Forslund SK, Cook H, et al. eggNOG 5.0: a hierarchical, functionally and phylogenetically annotated orthology resource based on 5090 organisms and 2502 viruses. Nucleic Acids Res. 2019;47: D309–D314. doi: 10.1093/nar/gky1085 30418610 PMC6324079

[65] Mistry J, Chuguransky S, Williams L, Qureshi M, Salazar GA, Sonnhammer ELL, et al. Pfam: The protein families database in 2021. Nucleic Acids Res. 2021;49: D412–D419. doi: 10.1093/nar/gkaa913 33125078 PMC7779014

[66] Johnson M, Zaretskaya I, Raytselis Y, Merezhuk Y, McGinnis S, Madden TL. NCBI BLAST: a better web interface. Nucleic Acids Res. 2008;36: W5–W9. doi: 10.1093/nar/gkn201 18440982 PMC2447716

[67] Edgar RC. MUSCLE: multiple sequence alignment with high accuracy and high throughput. Nucleic Acids Res. 2004;32: 1792–1797. doi: 10.1093/nar/gkh340 15034147 PMC390337

[68] Waterhouse AM, Procter JB, Martin DMA, Clamp M, Barton GJ. Jalview Version 2—a multiple sequence alignment editor and analysis workbench. Bioinformatics. 2009;25: 1189–1191. doi: 10.1093/bioinformatics/btp033 19151095 PMC2672624

[69] Bateman A, Martin M-J, Orchard S, Magrane M, Agivetova R, Ahmad S, et al. UniProt: the universal protein knowledgebase in 2021. Nucleic Acids Res. 2021;49: D480–D489. doi: 10.1093/nar/gkaa1100 33237286 PMC7778908

[70] Kumar S, Stecher G, Li M, Knyaz C, Tamura K. MEGA X: Molecular evolutionary genetics analysis across computing platforms. Battistuzzi FU, editor. Mol Biol Evol. 2018;35: 1547–1549. doi: 10.1093/molbev/msy096 29722887 PMC5967553

[71] Kelley LA, Mezulis S, Yates CM, Wass MN, Sternberg MJE. The Phyre2 web portal for protein modeling, prediction and analysis. Nat Protoc. 2015;10: 845–858. doi: 10.1038/nprot.2015.053 25950237 PMC5298202

[72] Jumper J, Evans R, Pritzel A, Green T, Figurnov M, Ronneberger O, et al. Highly accurate protein structure prediction with AlphaFold. Nature. 2021;596: 583–589. doi: 10.1038/s41586-021-03819-2 34265844 PMC8371605

[73] Pettersen EF, Goddard TD, Huang CC, Meng EC, Couch GS, Croll TI, et al. UCSF ChimeraX: Structure visualization for researchers, educators, and developers. Protein Science. 2021;30: 70–82. doi: 10.1002/pro.3943 32881101 PMC7737788

