# Supplementary figures and images for: Activation of ChvG-ChvI regulon by cell wall stress confers resistance to β-lactam antibiotics and initiates surface spreading in Agrobacterium tumefaciens
Source: PLoS Genet. 2022 Dec 8;18(12):e1010274. doi: 10.1371/journal.pgen.1010274 (PMC9731437; doi:10.1371/journal.pgen.1010274)

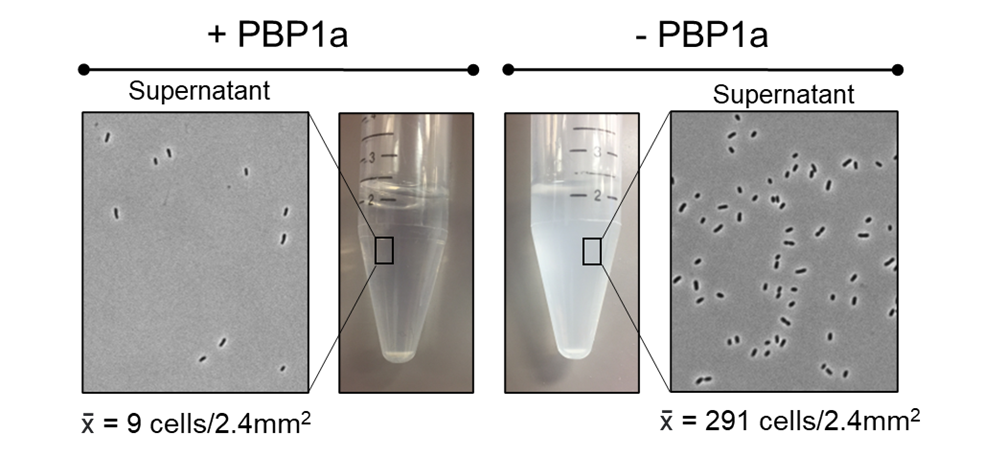

Supplement: S1 Fig — Conical tubes show turbidity after pelleting cells grown for 16 hours in PBP1a replete (+PBP1a) or depleted (-PBP1a) conditions. Cells were centrifuged at 1690 x g (3000 rpm in TX-400 rotor in a Sorvall Legend X1R centrifuge) for 10 minutes. Supernatants were spotted on a 1.25% ATGN agarose pad. x¯ = average number of cells from 10 fields of view. (TIF) [file pgen.1010274.s003.tif]

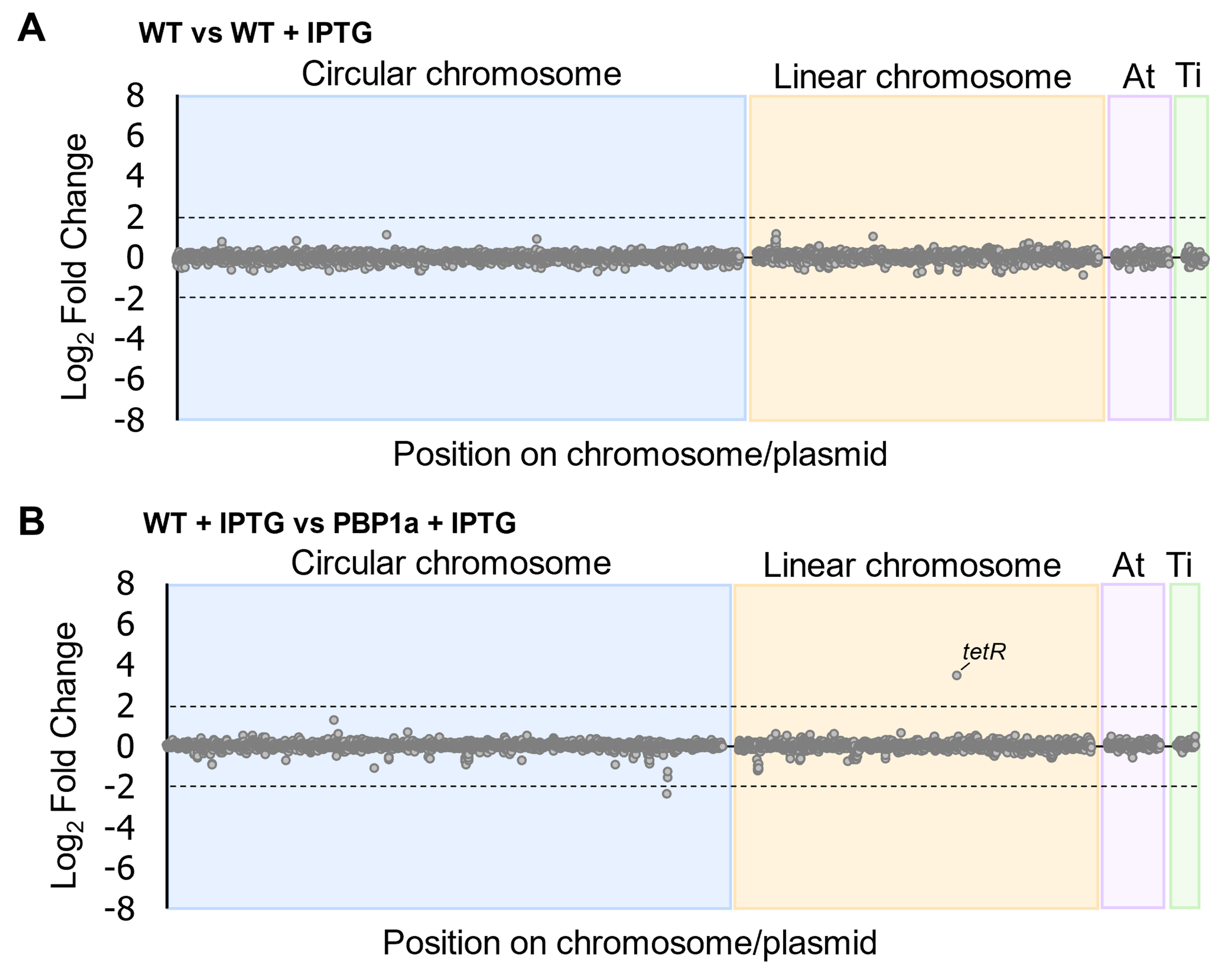

Supplement: S2 Fig — A. Plots comparing Log2Fold Change of the WT 6-hour transcriptome to that of the WT +ITPG 6-hour transcriptome. Gray dots represent a single transcript, and the dotted lines represent +/- 2.0 Log2Fold Change threshold. Plots are delimited by chromosomes and mega plasmids. B. Plots comparing Log2Fold Change of the WT +IPTG transcriptome to that of the PBP1a depletion strain with ITPG present to drive PBP1a expression. Comparisons shown are of the 6-hour transcriptomes. Gray dots represent a single transcript, and the dotted lines represent +/- 2.0 Log2Fold Change threshold. Plots are delimited by chromosomes and mega plasmids. (TIF) [file pgen.1010274.s004.tif]

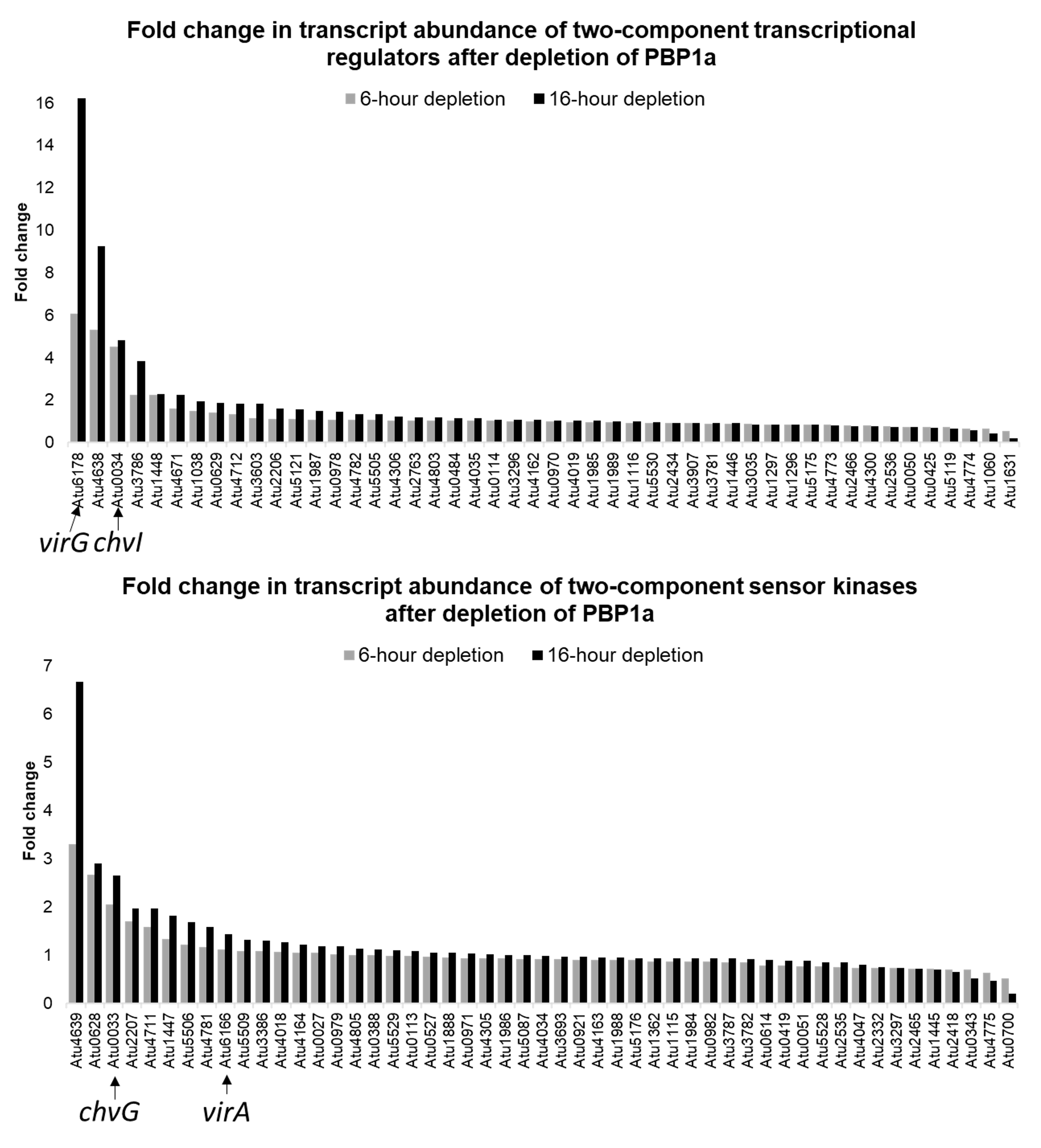

Supplement: S3 Fig — The fold change in expression level of TCS regulators and kinases are shown following 6 hours (gray) and 16 hours (black) of PBP1a depletion. The virAG and chvGI TCS pairs are labeled. (TIF) [file pgen.1010274.s005.tif]

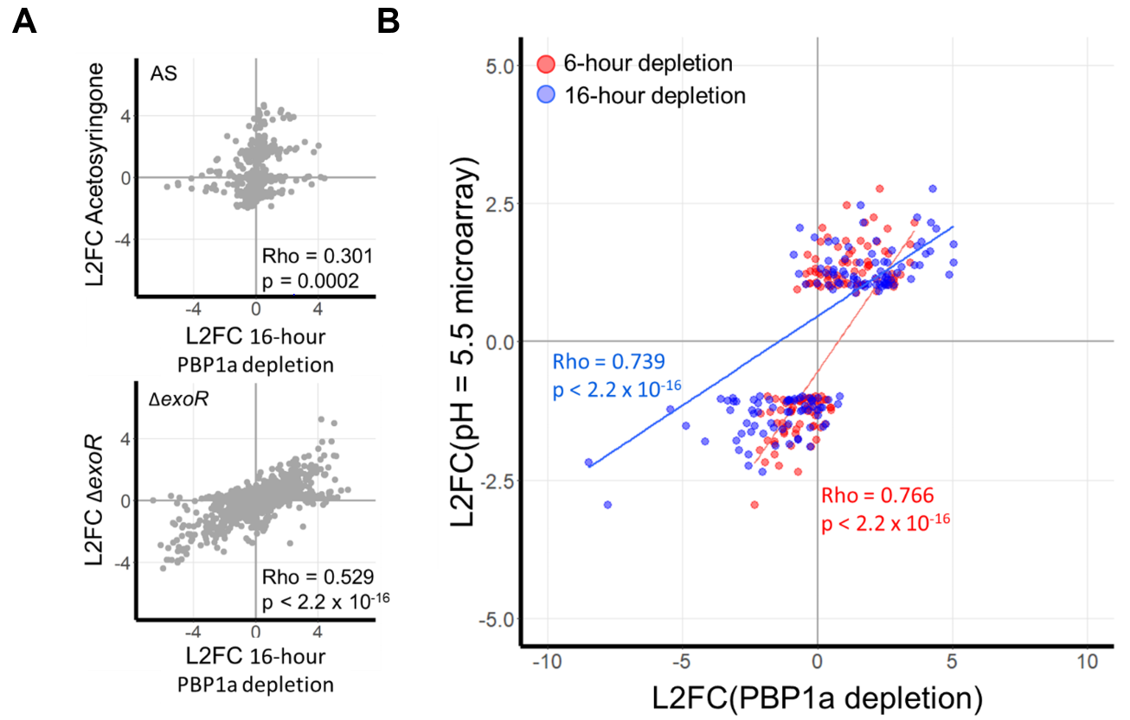

Supplement: S4 Fig — A. Correlation scatterplots depicting relationships between the log2fold-change (L2FC) values in the 16-hour PBP1a depletion and transcriptomic data sets taken under simulated virulence-inducing conditions (AS) and under simulated host-invading conditions (ΔexoR). Each point represents a unique transcript. AS, acetosyrinogone; Rho, Spearman correlation coefficient. B. Correlation scatterplots comparing L2FC values of transcripts in the pH 5.5 microarray, a condition known to induce the chvG-chvI regulon, to either the 6-hour (red) or 16-hour (blue) PBP1a depletion. Rho, Spearman correlation coefficient. (TIF) [file pgen.1010274.s006.tif]

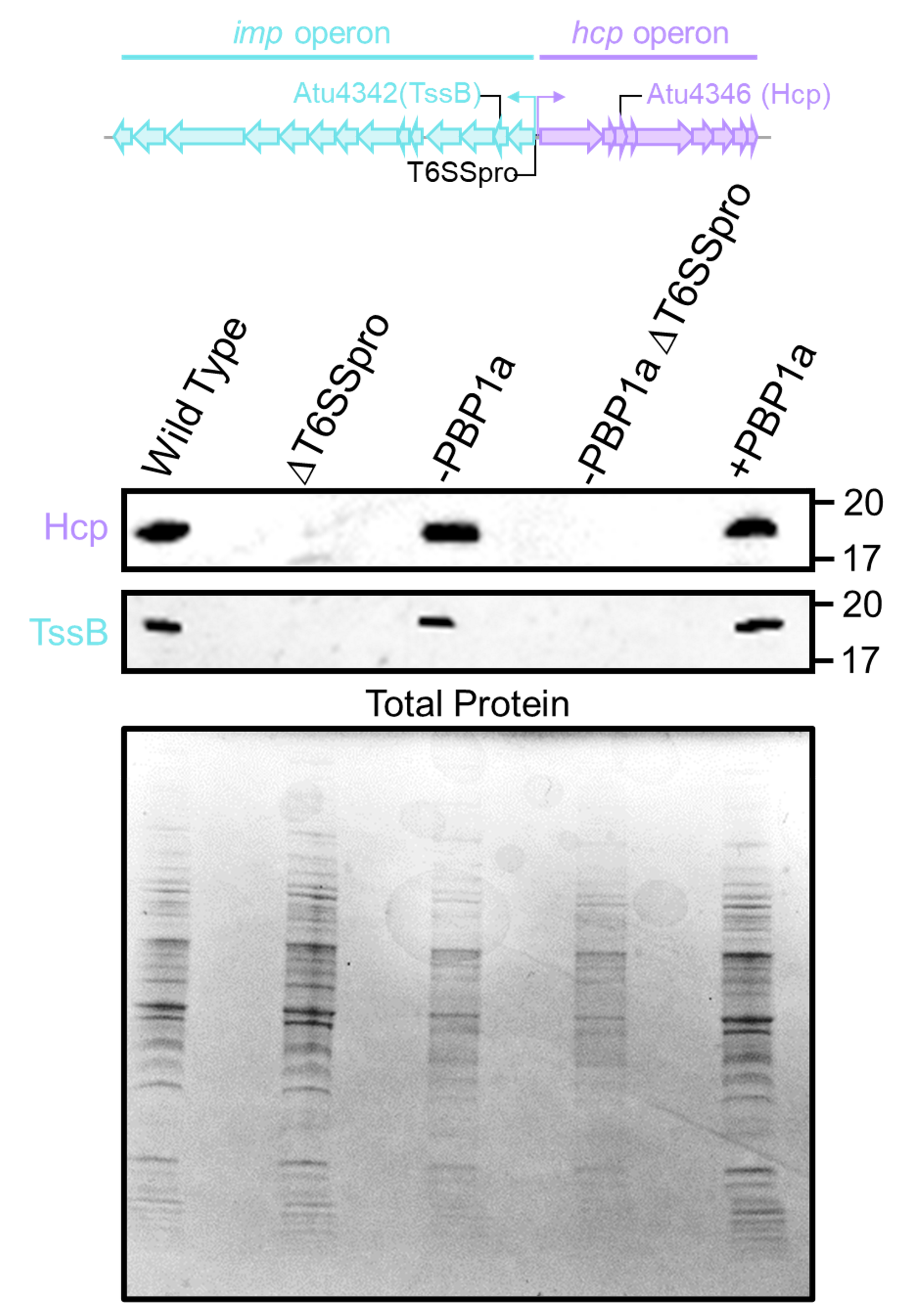

Supplement: S5 Fig — Top panel, diagram of the two operons encoding elements of Type VI Secretion in A. tumefaciens. T6SSpro labels the intergenic gap that is deleted in ΔT6SSpro strains. Middle panel, western blots using anti-Hcp and anti-TssB in each of the indicated strains. Protein sizes (kDa) are shown on the right. Bottom panel, Coomassie stained gel showing total protein from each strain. Western blots were performed as described in the methods with the following modifications. Lysates were prepared by pelleting cells via centrifugation and resuspending in 1X loading buffer. Next, the suspension was run through a 20G needle for lysis. 3 gels were loaded with identical concentrations of sample. BlueStain2 Protein ladder (P008-500) was loaded into the first well of each. One of the three gels was Coomassie stained at room temperature with gentle shaking for 10–15 minutes and imaged for total protein content. The other two gels were transferred to immobilon-FL transfer membranes, blocked with 0.5% milk, and transferred to a solution of TBS + 0.05% Tween 20 with 4 μL of 1:1000 dilution of either anti-TssB or anti-Hcp for 1 hour. Membranes were washed and transferred to TBS + 0.05% Tween 20 with 4 μL of 1:1000 dilution of anti-rabbit HRP goat IgG for 1 hour. (TIF) [file pgen.1010274.s007.tif]

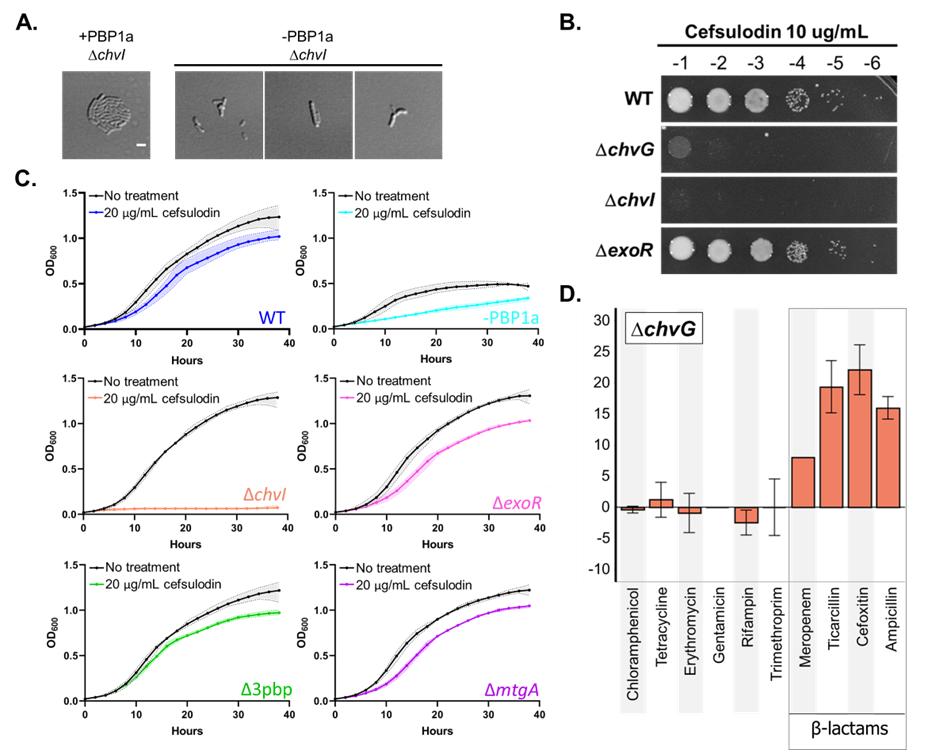

Supplement: S6 Fig — A. Micrographs of PBP1a depletion ΔchvI with (+PBP1A) or without (-PBP1A) IPTG. Cells were grown to exponential phase in ATGN media containing IPTG, spotted on an ATGN agarose pad with or without IPTG, allowed to grow for 16 hours, and imaged by DIC microscopy. B. Cell viability of each wild type, ΔchvG, ΔchvI, and ΔexoR spotted on an ATGN agar plate containing 10 μg/mL of cefsulodin. Ten-fold serial dilutions are indicated. C. Growth curves of WT, -PBP1a, ΔchvI, and ΔexoR, Δpbp3, ΔmtgA in the absence (black line) and presence of 20 μg/mL cefsulodin (colored line). D. Graph depicting the change in zone of inhibition from wildtype in ΔchvG against ten different antibiotic disks. Error bars represent +/- 1 standard deviation from the mean. (TIF) [file pgen.1010274.s008.tif]

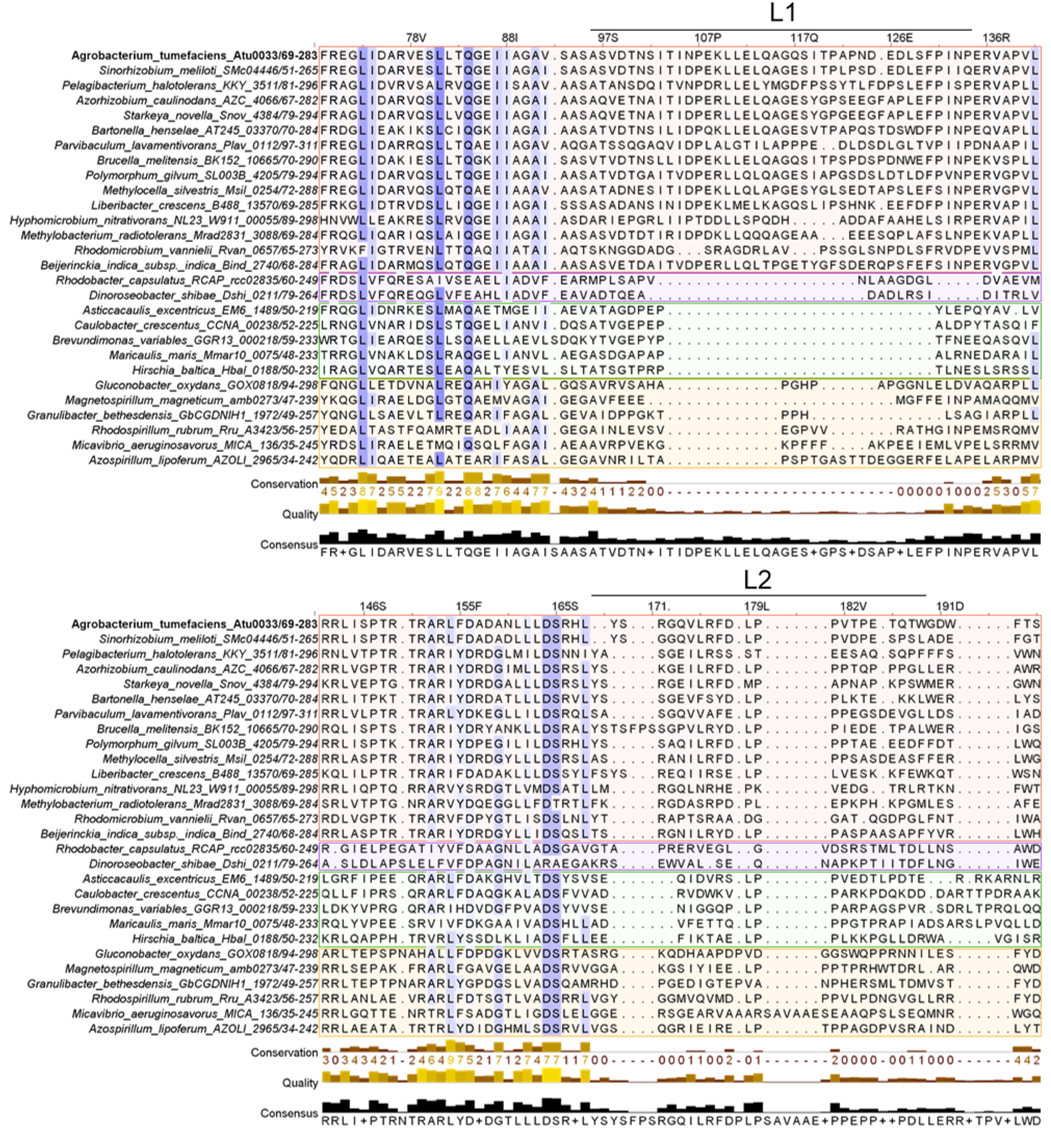

Supplement: S7 Fig — Partial MUSCLE alignment of ChvG ortholog periplasmic domains. Highlighted columns represent strong conservation across aligned sequences. Atu0033 (ChvG of A. tumefaciens) is the reference sequence for this analysis. L1 and L2 correspond to two conserved structural loops. Conservation, quality, and consensus scores for each site are represented as bar graphs under the alignment. Shading indicates Order of the bacterium containing the ChvG ortholog: Orange, Rhizobiales; Purple, Rhodobacterales; Green, Caulobacterales; Gold, Rhodospirales. (TIF) [file pgen.1010274.s009.tif]

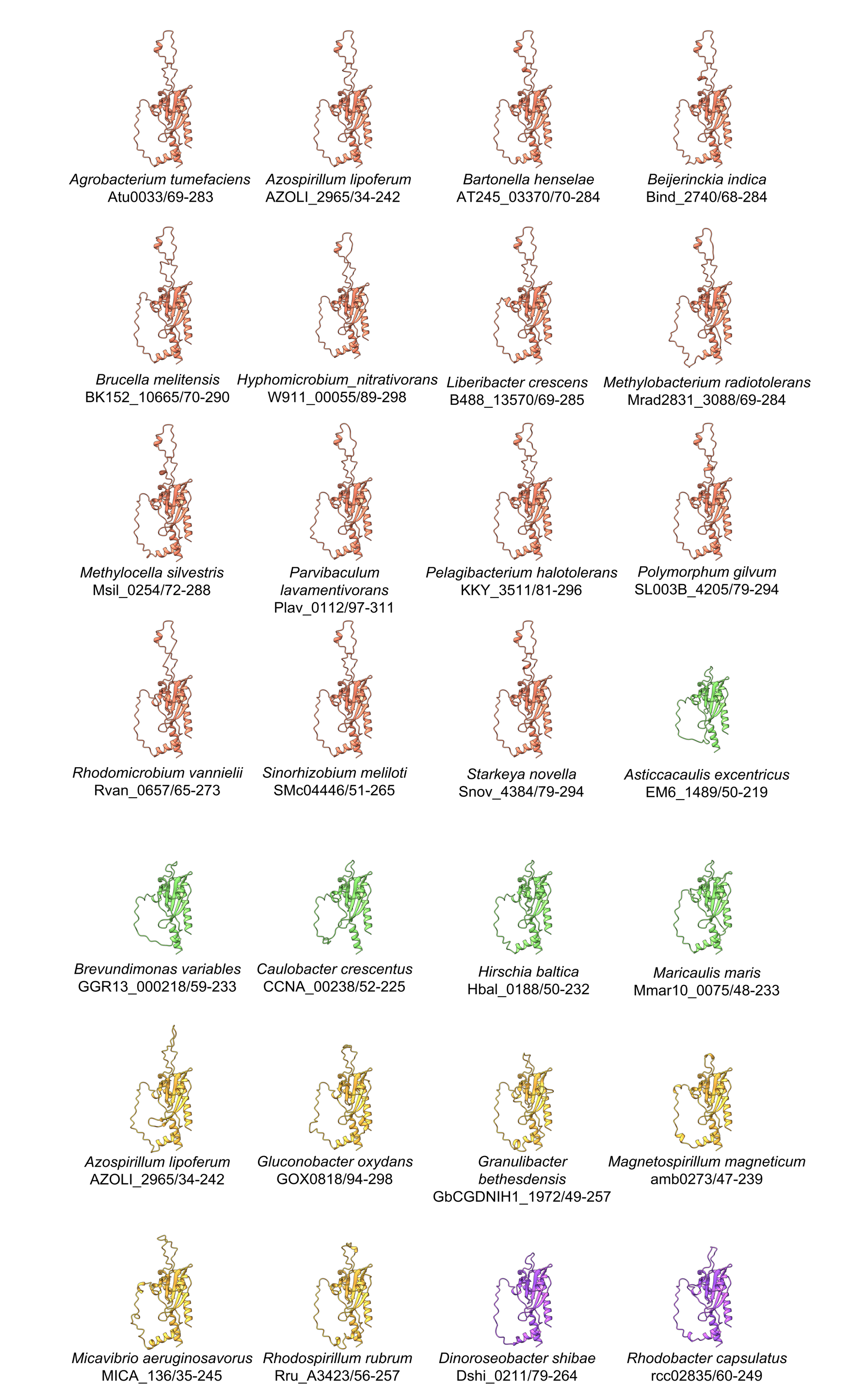

Supplement: S8 Fig — Phyre2 structural predictions for each organism displayed in Fig 6A of this work. Genus and species names as well as locus tags for each ChvG ortholog are provided. Range of numbers following the back slash are the amino acid sites used in structure prediction. Colors indicates order of the bacterium containing the ChvG ortholog: Orange, Rhizobiales; Purple, Rhodobacterales; Green, Caulobacterales; Gold, Rhodospirales. (TIF) [file pgen.1010274.s010.tif]

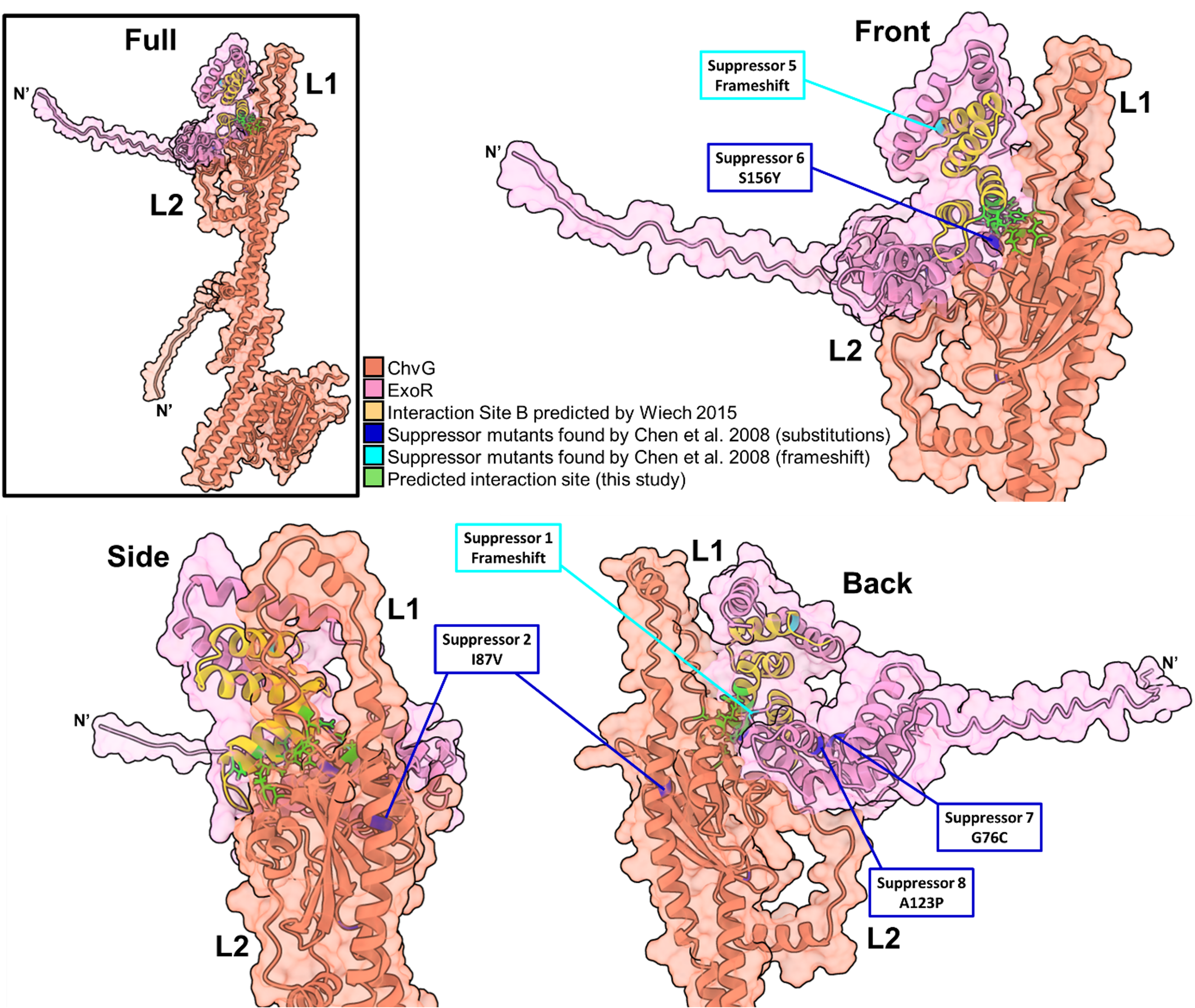

Supplement: S9 Fig — Previously characterized suppressor mutants (Chen et al. 2008) or predicted interaction sites (Wiech et al. 2014) are mapped onto the ExoR-ChvG AlphaFold Multimer structure presented here. The full structure is shown at the top left with detailed views of the ExoR-ChvG interface shown as the protein complex is rotated (front, side, and back). (TIF) [file pgen.1010274.s011.tif]

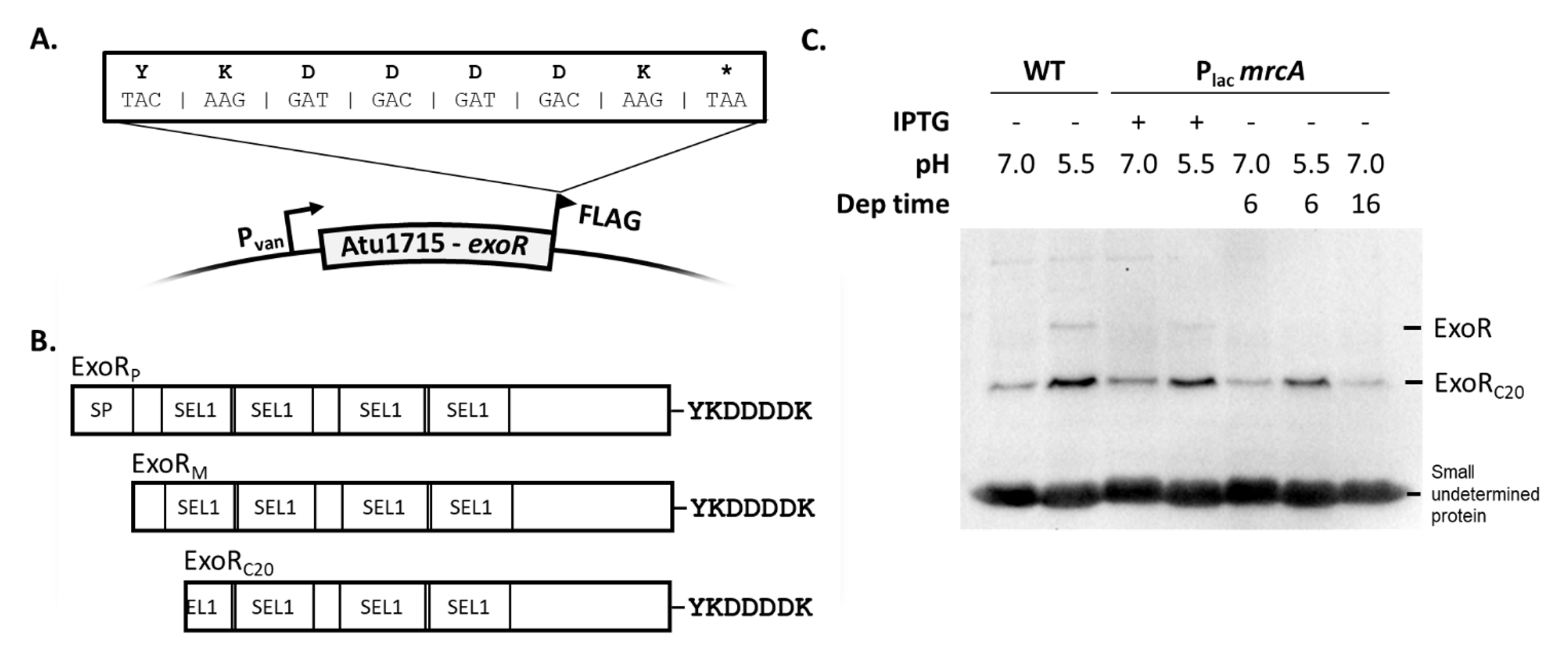

Supplement: S10 Fig — A. Schematic of Pvan driven expression of a ExoR-FLAG fusion protein. The sequence of the FLAG tag is shown above. The plasmid was introduced in the WT and PBP1a depletion strains to enable monitoring of ExoR proteolysis. B. Potential ExoR products are shown, including the pro-ExoR (ExoRP), mature ExoR (ExoRM), and cleaved ExoR (ExoRC20). ExoRP and ExoRM were indistinguishable and therefore denoted as ExoR. C. Western blot demonstrating ExoR-FLAG cleavage following acid treatment but not PBP1a depletion. Bands corresponding to ExoRC20 are labeled. Resolution of the ExoR on the blot does not allow for distinguishing between ExoRP and ExoRM. (TIF) [file pgen.1010274.s012.tif]

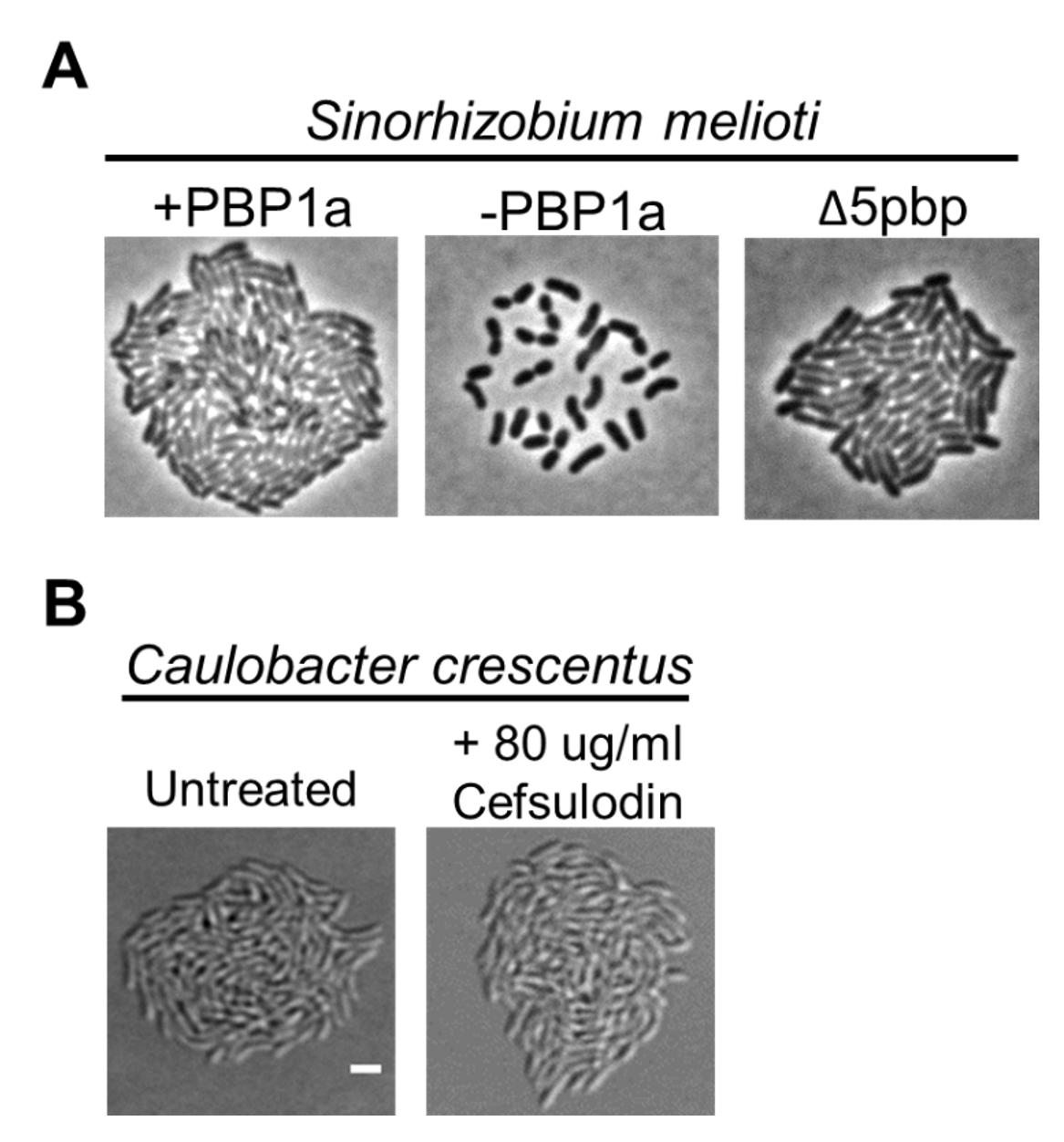

Supplement: S11 Fig — A. Micrographs of Sinorhizobium meliloti Rm2011 PBP1a replete, PBP1a depleted, and a strain with deletions of genes encoding all other high molecular weight PBPs (Δ5pbp). Each strain was grown to exponential phase, spotted on a 1% TY agar pad containing 1mM IPTG if inducing mrcA, allowed to grow for 16 hours, and imaged with phase microscopy. B. Micrographs of WT C. crescentus cells growth with or without cefsulodin. Cells were grown to exponential phase in PYE media, spotted on a PYE agar pad with or without cefsulodin, allowed to grow for 16 hours, and imaged by DIC microscopy. All scale bars depict 2 μm. (TIF) [file pgen.1010274.s013.tif]
